# Chinese Herbal Extracts Exert Neuroprotective Effect in Alzheimer’s Disease Mouse Through the Dopaminergic Synapse/Apoptosis Signaling Pathway

**DOI:** 10.3389/fphar.2022.817213

**Published:** 2022-02-28

**Authors:** Qianqian Huang, Chen Zhang, Sihao Qu, Shi Dong, Qihong Ma, Ying Hao, Zimin Liu, Shanglong Wang, Haibin Zhao, Yuanyuan Shi

**Affiliations:** ^1^ Third Affiliated Hospital, Beijing University of Chinese Medicine, Beijing, China; ^2^ School of Life Sciences, Beijing University of Chinese Medicine, Beijing, China; ^3^ Department of Pathology and Laboratory Medicine, Weill Cornell Medicine, New York, NY, United States; ^4^ Chenland Nutritionals, Irvine, CA, United States; ^5^ Dong Fang Hospital, Beijing University of Chinese Medicine, Beijing, China; ^6^ Shenzhen Research Institute, Beijing University of Chinese Medicine, Shenzhen, China

**Keywords:** Alzheimer’s Disease (AD), GPCRAC extracts, quantitative proteomics, scopolamine-treated model mice, dopaminergic synapse, apoptosis signaling pathway

## Abstract

**Background:** Alzheimer’s disease (AD) as an age-related, irreversible neurodegenerative disease, characterized by cognitive dysfunction, has become progressively serious with a global rise in life expectancy. As the failure of drug elaboration, considerable research effort has been devoted to developing therapeutic strategies for treating AD. TCM is gaining attention as a potential treatment for AD. *Gastrodia elata* Blume, *Polygala tenuifolia* Willd., Cistanche deserticola Ma, Rehmannia lutinosa (Gaertn.)DC., *Acorus gramineus* Aiton, and Curcuma longa L. (GPCRAC) are all well-known Chinese herbs with neuroprotective benefits and are widely used in traditional Chinese decoction for AD therapy. However, the efficacy and further mechanisms of GPCRAC extracts in AD experimental models are still unclear. The purpose of this study was to investigate the synergistic protective efficacy of GPCRAC extracts (composed of extracts from these six Chinese medicines), and the protein targets mediated by GPCRAC extracts in treating AD.

**Methods:** Scopolamine-induced cognitive impairment mouse model was established to determine the neuroprotective effects of GPCRAC extracts *in vivo*, as shown by behavioral tests and cerebral cholinergic function assays. To identify the potential molecular mechanism of GPCRAC extracts against AD, label-free quantitative proteomics coupled with tandem mass spectrometry (LC-MS/MS) were performed. The integrated bioinformatics analysis was applied to screen the core differentially expressed proteins in vital canonical pathways. Critical altered proteins were validated by qPCR and Western blotting.

**Results:** Administration of GPCRAC extracts significantly recovered scopolamine-induced cognitive impairment, as evidenced by the improved learning and memory ability, increased Ach content and ChAT activity, as well as decreased AchE activity in the hippocampus of mice. In total, 390 proteins with fold-change>1.2 or <0.83 and *p* < 0.05 were identified as significant differentially expressed proteins, of which 110 were significantly up-regulated and 25 were significantly down-regulated between control and model group. By mapping the significantly regulated proteins, we identified five hub proteins: PPP2CA, Gsk3β, PP3CC, PRKACA, and BCL-2 that were associated with dopaminergic synapse and apoptosis signaling pathway, respectively. Western blotting and QPCR demonstrate that the expression levels of these core proteins could be significantly improved by the administration of GPCRAC extracts. These pathways and some of the identified proteins are implicated in AD pathogenesis.

**Conclusion:** Administration of GPCRAC extracts was effective on alleviating scopolamine-induced cognitive impairment, which might be through modulation of dopaminergic synapse and apoptosis signaling pathway. Consequently, our quantitative proteome data obtained from scopolamine-treated model mice successfully characterized AD-related biological alterations and proposed novel protein biomarkers for AD.

## 1 Introduction

Alzheimer’s disease (AD) is a progressive, irreversible neurodegenerative disease characterized by learning decline and memory dysfunction with neuropathologically negative consequences (Feng et al., 2020, Tan, Wu, Dong et al., 2018, and Zhang, 2020). Epidemiological investigations suggest that the number of people with dementia in the world may reach 131.5 million in 2050 (Gavrilova & Alvarez, 2021). However, there is currently no disease-modifying therapies available for AD (Liu et al., 2020). The cost-intensive development of drugs that target a single protein may not achieve expected therapeutic results. Traditional Chinese medicine associated with multiple targets and various pharmacological actions have obvious advantages in treating AD.

The combination of Gastrodia elata Blume,Polygala tenuifolia Willd*.*, Cistanche deserticola Ma, Rehmannia lutinosa (Gaertn.)DC., Acorus gramineus Aiton and Curcuma longa L.(GPCRAC) is commonly used in Chinese medicinal prescription for AD therapy, such as Tianma Xingnao capsule, which has been licensed for clinical use in China and widely used to treat headaches, chronic pain, fatigue, memory loss and insomnia (Youli et al., 2013; Yaming et al., 2021). Several active ingredients extracted from these six traditional Chinese medicines have shown neuroprotective properties. Curcuma, the primary ingredient of Curcuma longa L., have receives a lot of attention for its promising neuroprotective effects in the prevention and treatment of AD (Chainoglou & Hadjipavlou-Litina, 2020). Gastrodin, the major functional component of the orchid plant *Gastrodia elata* Blume, is widely recognized for its vast variety of biological functions. Meng et al. reported that Gastrodin, has the potential to promote hippocampus neurogenesis by inhibiting pro-inflammatory cytokines generated by oligomer-induced A42 in a dose-dependent manner (M. Li & Qian, 2016). Additionally, Gastrodin was able to reduce the abnormal expression levels of synaptic proteins (Xiao et al., 2016). The main active ingredients of *Polygala tenuifolia* Willd. include oligosaccharide esters (3,6′-disinapoyl sucrose, Sibiricose A5, Sibiricose A6), Onjisaponin, and Xanthones (She et al., 2011), which have been documented to possess mitigative effects on neurotoxicity. Previous study have indicated that Onjisaponin B could contribute to the restoration of cognitive ability by improving the antioxidant capacity in D-gal induced aging rats (Li et al., 2018). Furthermore, Ethnocide, a physiologically active component isolated from *Cistanche deserticola* Ma, can help with learning and memory loss by modulating hippocampus insulin, glucose transport, and energy metabolism (Dai et al., 2020). Also, it was previously shown that Acteoside, which is found in *Cistanche deserticola* Ma, can ameliorate the deficiency of learning ability in mice induced by scopolamine (Lee, Jeong, Lee, & Kim, 2006). Taken together, these active components isolated from these Chinese herbs may exert synergistic action against AD through multiple targets and pathways. Moreover, the combination of extracts derived from these six Chinese medicines have been developed as an adjunct to dietary supplements for treating AD (CuralUtra, Batch No. CM18220191028-2, Product code: T-4010-1). It is also applied for a patent of novel formula to improve cognitive impairment (patent number: 201911113149. X). Even though there are lots of evidence of therapeutic application of GPCRAC extracts in AD treatment, but it remains unknown whether the efficacy of the GPCRAC extracts in AD experimental model and the further mechanism. And, the exploration of potential pharmacological mechanisms is still a challenge in the research of TCMs containing multiple herbal constituents. The powerful proteomics technology is now pushing forward the frontiers of Chinese medicine compound mechanism research. Quantitative proteomics based on Mass Spectrometer (MS) has been shown to be a powerful technique for elucidating novel pathophysiological processes and possible therapeutic targets, allowing for in-depth analysis of protein expression variations. Through proteomics analysis, we assessed the possible protective effect of GPCRAC extracts in scopolamine-induced model mice and investigated the underlying mechanism.

## 2 Materials and Methods

### 2.1 Drugs and Reagents

These herbal extracts of GPCRAC were purchased from Shanxi Jiahe Biotechnology Co., Ltd. (Shanxi China). Scopolamine hydrobromide was purchased from Beijing Bailingwei Technology Co., Ltd., (Beijing China). Batch No. LI10Q53. Huperzine A (70 mg/tablet) was purchased from Henan Tai long Pharmaceutical Co., Ltd., (Henan China). Batch No.190202. Acetylcholine (Ach) assay kit, Choline acetyltransferase (ChAT) assay kit and Acetylcholinesterase (AchE) assay kit was purchased from Nanjing Jian Cheng Bioengineering Institute (Jiangsu, China).

### 2.2 Preparation of GPCRAC Extracts

GPCRAC extracts were prepared by Gastrodia elata Blume extract standardized to 1% Gastrodin (Batch No. C7M-A-803456), Polygala tenuifolia Willd extract standardized to 1% 3, 6′-Disinapoly sucrose (Batch No.CY2-A-808331), Acorus gramineus Aiton extract (10:1) (Batch No.CSCP-A-710215), Cistanche deserticola Ma extract standardized to 20% phenylethanol glycosides (Batch No.190501), Rehmannia lutinosa (Gaertn.)DC.extract (5:1) (Batch No. CSDH-A-900106), Curcuma longa L.extract standardized to 95% total curcuma (Batch No. CJH-A-908220) at the ratio of 1.25:0.67:0.18:0.3:0.125:0.1 (The ratio is based on the calculation of pharmacopoeia recommended dosage and extracts rate). The ratio of Curcuma longa L. extract used the low daily dose of curcumin recommended by the Chinese Pharmacopoeia (the recommended dosage is 200 mg). The phytochemical standardization and HPLC characteristic fingerprint for each herbal extract was completed by Shanxi Jiahe Biotechnology Co., Ltd., (Shanxi China) (see Supplementary Materials). Briefly, the crude drugs of *Gastrodia elata* Blume, *Polygala tenuifolia* Willd, Cistanche deserticola Ma were pulverized to powder and sieved through a 20-mesh sieve. *Gastrodia elata* Blume was extracted three times with 70% ethanol for 2 h each time. *Polygala tenuifolia* Willd was extracted three times with 80% ethanol for 2 h each time. Cistanche deserticola Ma was extracted three times with 50% ethanol for 2, 1.5, 1.5 h, with a liquid-to-material ratio of 6, 5, 4, respectively. Curcuma longa L. was extracted two times with 95% ethanol for 2 h each time. *Acorus gramineus* Aiton was soaked with 8-fold of water for 3h, and then extracted three times, each time for about 2 h. Rehmannia lutinosa (Gaertn.)DC. was soaked with 8-fold of water for 1 h and extracted three times for 2, 2, 1 h, respectively. These extracts were then filtered, condensed under vacuum, freeze-dried into powder.

### 2.3 LC-MS Analysis of GPCRAC Extracts

Dionex Ultimate 3000 UHPLC system equipped with a binary pump, an autosampler, a solvent degasser, and a thermostatic column compartment (Thermo Fisher, Waltham, MA, United States) were used for analysis component of GPCRAC extracts. All chromatographic separations were performed on a BDS HYPERSIL C18 column (150 mm × 2.1 mm, 2.4 μm; Thermo Fisher Scientific). The mobile phase consisted of acetonitrile (A) and 0.1% formic acid aqueous solution at a flow rate of 0.3 ml/min, with gradient elution as follows: 0∼45 min, 95%B; 45.0–45.1 min, 25%B; 45.1–50 min,95%B. And the column temperature was set to 45°C. Mass Spectrometer (MS) detection was performed on a Q Exactive™ Plus mass spectrometer (Thermo Fisher Scientific, United States) equip ed with an electrospray ionization source (ESI) using a positive ion spray voltage of 3.5 k eV and a negative ion spray voltage of 3.0 k eV. The mass spectrometry conditions were as follows: the sheath gas and auxiliary gas was both high-purity nitrogen (purity>99.99%); the flow rates were 40 arbitrary units and 20 arbitrary units respectively; the capillary temperature was 320°C; the sheath gas flow rate was 35.0 μl/min, the aux gas flow rate was 10 μl/min, the sweep gas flow rate was 10 μl/min; The mass scanning range was m/z 120∼1800.

### 2.4 Animals and Treatment

102 SPF male C57BL/6J mice (weight 20 ± 2 g) were used in our study. All animals were produced by Jinan Pengyue Experimental Animals Education Co., Ltd (Beijing, China). License number: SCXK (Lu) 2018-0,003.6. The feeding procedures of animals and experimental operations have been approved by the Animal Care and Use Committee of Beijing University of Chinese Medicine (BUCM-4-2019102105-4119). Mice were housed in cages and kept under standard breeding conditions (12:12 h light/dark cycle, controlled room temperature (23 ± 2°C), sanitary conditions, standard diets, and stress-free environment. After 1 week of accommodation, mice were randomly divided into six equal-sized groups: control group, model group (scopolamine hydrobromide of 3 mg/kg/day), HupA group (HuperzineA, positive drug), GPCRAC-L group (GPCRAC extracts of 128 mg/kg/day), GPCRAC-M group (GPCRAC extracts of 256 mg/kg/day), GPCRAC-H group (GPCRAC extracts of 512 mg/kg/day), every seventeen mice were divided into one group. The administration dosage of GPCRAC extracts was calculated using dosage conversion relationship between mice and human. All mice were administered intragastrically for 30 consecutive days. Behavioral tests were then performed to examine mouse cognitive deficits at 31, 36, 40 days, respectively. One hour after GPCRAC extracts treatment, behavioral tests were conducted. Mice were administrated intraperitoneally with scopolamine (3 mg/kg) 15–20 min before behavioral test. After the last behavioral test, mice were euthanized by cervical dislocation, the hippocampus tissues were isolated and collected on ice and then stored in a −80°C cryo-freezer for label-free shotgun proteomics analysis and bioinformatics to provide insights on the protein responses induced by GPCRAC extracts in the brain of AD mice.

### 2.5 Behavioral Tests

#### 2.5.1 Novel Object Cognition Test

Novel Object Recognition (NOR) test was performed to detect the short-term memory ability of mice. When contacting a new object for the first time, mice will explore more frequently instead of spending time on an object they have seen before (familiar object). On the first day, place two identical objects in a room and allow the mouse to explore for 5 min.24 h later, one of the objects was replaced by a novel object in the chamber. Exploration is defined as mice touching or sniffing the object within a distance ≤2 cm from the object (Panta et al., 2020). Recording the Total Exploration Time of Novel Object (Tn)and The Total Exploration Time of Familiar Object (TF) within 5 min. Calculate the Recognition Index: RI = TN/(TN + TF) (Wang et al., 2016). After each mouse experiment is over, clean the open field box with 75% ethanol.

#### 2.5.2 The Step-Down Passive Avoidance Test

The step-down passive avoidance (SDA) test consists of a rectangular plexiglass inner box with parallel steel rods on the grid floor and a wooden platform. During the training phase, the animal was placed on the grid floor, and then the power was turn on, the electric shock lasted 5 min. After 24 h, the animal was placed on the wooden platform while giving an electrical shock. The step-down latency (recorded as the time for the animals to jump-down from the platform) and the number of errors (the animals touching the grid floor with paw within 300 s) were used to evaluate learning and memory performance of mice. If the mouse does not jump off the platform within 300 s, the number of errors is recorded as 0 and the incubation period is recorded as 300 s.

#### 2.5.3 Morris Water Maze Test

The Morris water maze test was performed to calculate the ability of spatial learning and memory in AD mice. The test, which lasts for five consecutive days, was divided into two parts, the first part was the positioning navigation experiment in the first 4 days. On the fifth day, the platform was removed for space exploration experiment (Sun et al., 2020). During the training session, each mouse was placed into the water, facing to the wall of the pool, in one of the four quadrants. Simultaneously start with computer recording equipment. The swim time was set to 60 s. The time spent in searing platform is called escape latency, and swimming trajectories and swimming distance of the mice were recorded within the 60 s period. If the mouse failed to find the platform, alternatively, put the mouse on the platform and stand for another 15 s.The platform was removed on the last day (Chen et al., 2020).

### 2.6 Protein Extraction and Digestion

In this experiment, there were four groups of mice (*n* = 17 per group) to evaluate the proteome treated by GPCRAC extracts. In each group, nine hippocampus samples isolated from different animals were pooled into three samples for proteomic analyses, followed by protein extraction and quantification. The proteomics analysis protocol has been reported previously (Zhang L. et al., 2021; Hao et al., 2021). The proteins were extracted with the mammalian tissue total protein extraction kit (AP0601-50, Bang Fei Bioscience Co, Ltd, Beijing, China), and the protein concentration was determined using the protein quantification kit according to the manufacturer’s instructions. Approximately 25 ug of brain tissues was homogenized in 250 ul of extraction buffer for 2 min using a homogenizer. Then, the lysates were centrifuged at 20,000 × *g* for 10 min at 4°C. Next, the supernatant was recovered. The protein concentration was measured by BCA (bicinchoninic acid) kit (Thermo Fisher Scientific, America) according to the manufacturer’s protocol. After protein quantification, Protein solution samples were prepared (Hao et al., 2021). Then, the samples were digested with trypsin following the Filter Aided Sample Preparation (FASP) protocol. All the peptide samples were collected for mass spectrometry analysis.

### 2.7 Label Free Quantitative Proteomic Analyses

Each sample was separated using a nanoliter flow rate Easy nLC1000 system. Digested peptide mixtures were pressure-loaded onto a C18 trapping column (150 μm × 120 mm, 1.9 μm, Thermo Fisher Scientific, United States) and the column was washed with buffer A (water, 0.1% formic acid) and buffer B (80% acetonitrile and 0.1% formic acid) at a flow rate of 600 nL/min. After chromatographic separation, LC-MS/MS analysis was conducted using Q Exactive HF-X mass spectrometer (Thermo Scientific) that was coupled with Easy nLC (Thermo Fisher Scientific) for 88 min. The mass spectrometer was operated in positive ion mode with MS1 survey scan (m/z: 350–1,550). The resolution of the primary mass spectrum was 120,000, Automatic gain control (AGC) target was set to 3e6, and the maximum time of the primary ion was 20 ms. Normalized collision energy was 30 eV.

### 2.8 Mass Spectrometry Data Analysis and Bioinformatics Analysis

Peptide targeted identification was conducted with the SEQUST search engine, using sequences of human proteins downloaded from Uniprot (Zhang W. et al., 2021). All peptide raw files were analyzed by the Proteome Discover (2.2.0.388) and compared against the Uniprot Mus protein database (uniprot-Mus + musculus_20190102. fasta). Results were filtered with Peptide false discovery rate (FDR) ≤0.01. The following parameters were used in this analysis: Enzyme, Trypsin; Max Missed Cleavages, two; Peptide Mass Tolerance, ± 15 ppm; Fixed modifications, Carbamidomethyl (C); Variable modifications, Oxidation (M); Acetyl (Protein N-term); Fragment Mass Tolerance, 20 mmu; peptide confidence, high.

Then, Gene Ontology-term annotations and Kyoto Encyclopedia of Genes and Genomes (KEGG) pathways analysis using DAVID Bioinformatics Resources 6.8 (https://david.abcc.ncifcrf.gov/home.jsp) was performed. The protein–protein interaction (PPI) networks was constructed using STRING (https://www.stringdb.org), and the Cytohubba plugins of Cytoscape (version 3.6.0, Boston, MA, United States) was used to further analysis the PPI network. Principal Component Analysis (PCA), Orthogonal Partial Least Squares-Discriminant Analysis (OPLS-DA) model and Samples correlation heatmaps was constructed using MetaboAnalyst5.0 (https://www.metaboanalyst.ca/MetaboAnalyst/Secure/analysis/AnalysisView.xhtml). Partial Least-Squares Discriminant Analysis (PLS-DA) model and Permutation Test were performed with SIMCA-P software (version 14.1; Umetrics, Umea, Sweden). GraphPad Prism (version 8.0.2, San Diego, CA, United States) was used to visualize the volcano plot and box plots. Differential protein correlation graph used bioinformatics website(http://www.bioinformatics.com.cn/). The protein number map was generated using R software (version 3.3.3, Vienna, Austria). Pathway based data integration and visualization was performed using Pathview (https://pathview.uncc.edu/analysis).

### 2.9 Measurement of Acetylcholine Content, Acetylcholinesterase and Choline Acetyltransferase Activity From the Mice Brain

The hippocampus of mice was collected and homogenized with 10-fold volume of RIPA buffer. After centrifuge, the upper liquid was obtained and used to detect the content of Ach and the activities of ChAT and AchE in the hippocampus according to the manufacturer’s instructions of the Ach assay kit (CAT: A105-1-2), ChAT assay kit (CAT: A079-1-1) and AchE assay kit (CAT: A024-1-1).

### 2.10 RT-qPCR Analysis

Total RNA was extracted from hippocampus tissue using RNeasy^®^ Lipid Tissue Mini Kit (QIAGEN, Valencia, CA, United States). The quality and quantity of RNA were measured using spectrophotometer (Thermo Nano Drop™ 2000c, United States). We used the following Primers: Forward Primer-PPP2CA *TGG​GTT​CTA​CGA​CGA​GTG​TT*, Reverse Primer-PPP2CA Reverse Primer *AGA​AGA​TCT​GCC​CAT​CCA​CC*. Forward primer-PPP3CC *TGC​ACA​CAG​GAT​CCG​AAG​TT*, Reverse Primer-PPP3CC *CTT​TCG​GGG​TGG​CAT​TCT​CTC*. Forward Primer-Caspase-3 *CGG​AAT​TCG​AGT​CCT​TCT​CC*, Reverse Primer-Caspase-3 *AGC​ATG​GAC​ACA​ATA​CAC​GG.* Forward Primer-GAPDH *AGG​TTG​TCT​CCT​GCG​ACT​TCA*, Reverse Primer-GAPDH *TGG​TCC​AGG​GTT​TCT​TAC​TCC*. RNA (2 μg) was converted into complementary DNA using Reverse Transcription Master Mix (QIAGEN, Valencia, CA, United States). RT-qPCR was determined using SYBR Green Master mix on a CFX-96 system (QIAGEN, Valencia, CA, United States). We used the 2 ^ -delta Ct method for the quantification of samples. GAPDH gene was chosen as the endogenous control to normalize variance between samples.

### 2.11 Western Blotting

Total protein was extracted from frozen Brain tissue by RIPA Lysis Buffer (Applygen Technology Co., Ltd., Beijing, China, Catalog number: C1055) with protease inhibitor (100X) and a phosphatase inhibitor (100X). Protein concentration from tissue homogenates was measured with a BCA protein assay kit (Applygen, Beijing, China). 20 μg samples were separated by 8–12% SDS-PAGE, and then the protein was transferred to PVDF membranes (0.45 or 0.22 μm, Millipore, United States) and were blocked with 5% fat-free milk. After blocking for 2 h, the membranes were washed three times for 10 min per time by TBST. The membranes were incubated with indicated primary antibodies: anti-PP2A-alpha (ab106262,abcam, 1:1,000), phosphor-PKAC-*α* (D45D3, cell signaling technology, 1:500), PKAC-*α* (D38C6, cell signaling technology, 1:1,000), PPP3CC (ab154863,abcam, 1:1,000), Phospho-GSK3β (Ser9) (67558-1-Ig, proteintech, 1:3,000), GSK3β (22104-1-AP, proteintech, 1:3,000), BCL-2 (ab194583, abcam, 1:3,000), Bax (ab173026, abcam, 1;3,000) and GAPDH (ab8245, abcam, 1:4,000) overnight at 4 °C.Then the membranes incubated with primary antibodies goat anti-rabbit IgG or rabbit anti-mouse after washed three times for 2 h. ChemiDoc™ MP Imaging system (Bio-Rad Co, United States) and ImageJ software were used to quantify the protein bands.

### 2.12 Statistical Analysis

For comparisons between two groups, we used Student’s unpaired *t*-test. One-way ANOVA with post-hoc test was applied to determined significant differences for multiple groups. All data are shown as mean ± SE, and *p* ≤ 0.05 was considered.

## 3 Results

### 3.1 GPCRAC Extracts Ameliorated Scopolamine-Induced Learning and Memory Deficit

**FIGURE1:**
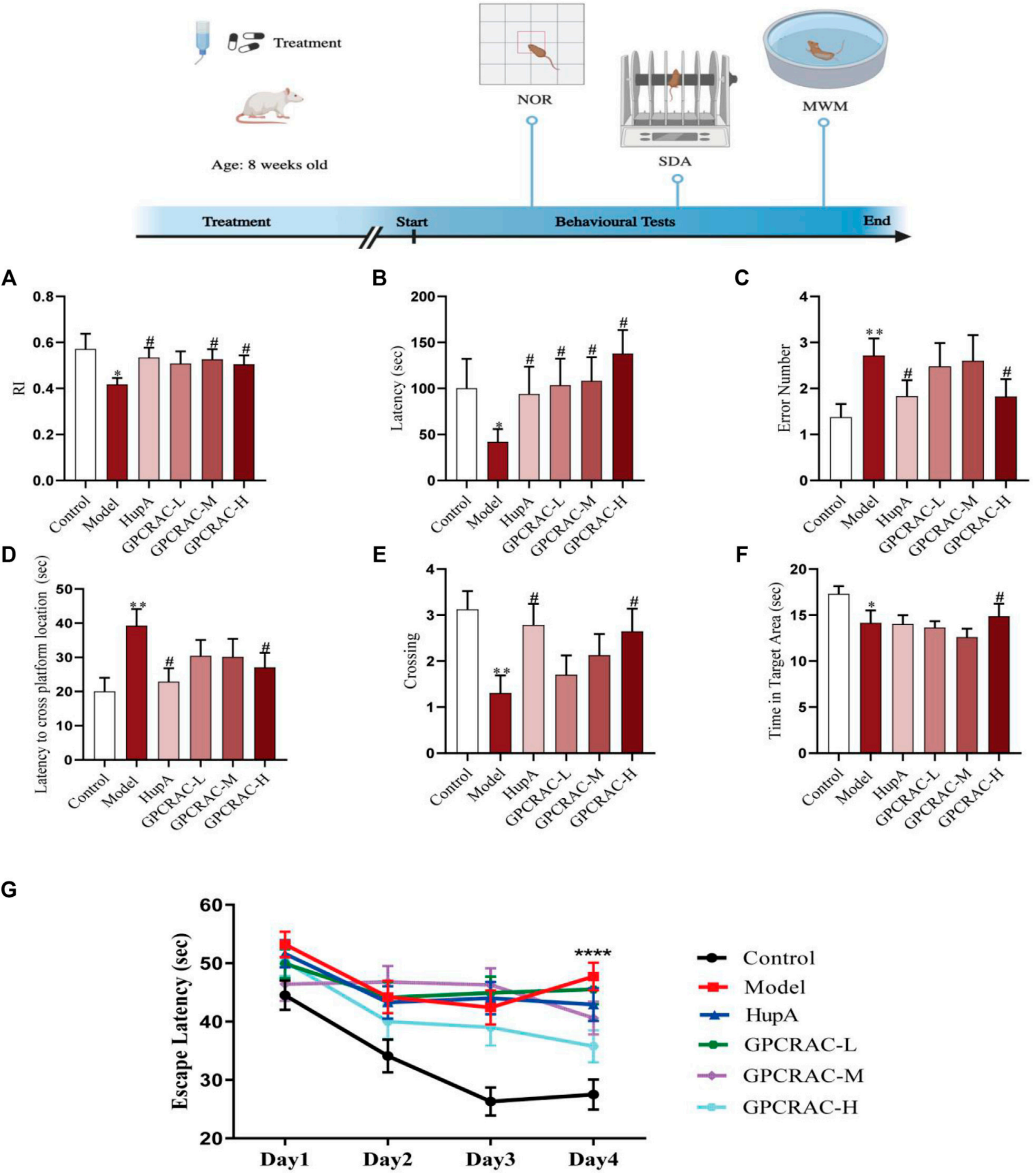
Ameliorating effect of GPCRAC extracts on learning and memory deficits in scopolamine-treated mice. The exploration time percentage of novel objects (Recognition Index RI) in novel object recognition abilities **(A)**; The latency and the number of errors in step-down passive avoidance test **(B,C)**; The escape latency to find hidden platform **(D)**, the numbers of crossing **(E)**, retention time spent in target quadrant **(F)** on the last day during Morris water maze; The escape latency during positioning navigation test (the first four consecutive days training) **(G)**. Data are expressed as mean ± SE (*n* = 17). *p < 0.05, **p < 0.01 and ***p < 0.001 compared to the control group; #*p* < 0.05, ##*p* < 0.01 and ###*p* < 0.001 compared to the scopolamine-treated group.

To assess the neuroprotective effect of GPCRAC extracts *in vivo*, a mouse model of scopolamine-induced cognitive impairment was created. Novel Object Recognition (NOR) test, Step-Down Passive Avoidance (SDA) test, and Morris Water Maze (MWZ) test were conducted to evaluate the neuroprotective of GPCRAC extracts on the scopolamine-induced learning and memory deficit in mice (Figure 1). In the NOR test, scopolamine-treated group (model group) exhibited low-level characteristics in recognize recognition index compared with control group, however, middle-dose group and high-dose group of GPCRAC extracts dramatically improved the reduced RI in AD mice (Figure 1A). By SDA test, we detected that the treatment of GPCRAC extracts notably recovered scopolamine-induced shorter step-down latency in a dose-dependent manner (Figure 1B) and GPCRAC extracts with high-dose group significantly reduced the number of errors in mice compared with model group (Figure 1C). In the Morris water maze (MWZ) test, the AD mice showed learning and memory deficits than that of control group, while GPCRAC extracts with high dose group improved cognitive impairment as evidenced by the decreased escape latency to find the platform site and more crossing numbers in platform area on the final day after removed the platform (Figures 1D,E). During the exploration period, decreased time of staying at target quadrant was also observed in scopolamine treated mice (Figure 1F). Additionally, the escape latency recorded during positioning navigation test revealed that AD mice took longer to reach the platform (escape latency) than the control and GPCRAC extracts group, with the effect most prominent on Day 4 (Figure 1G), indicating significant spatial learning and memory impairment after scopolamine exposure.

### 3.2 GPCRAC Extracts Ameliorated Scopolamine-Induced Hippocampal Neuron Damage

**FIGURE 2:**
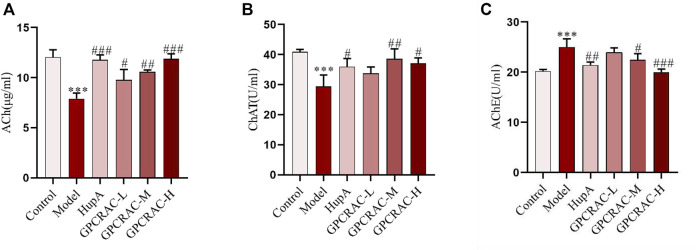
Ameliorating effect of GPCRAC extracts on cholinergic system dysfunction in hippocampus of scopolamine-induced cognitive deficit mice. Ach content **(A)**, ChAT activity **(B)** and AchE activity **(C)** in hippocampus were measured according to assay kit instructions; Data are expressed as mean ± SE. *p < 0.05, **p < 0.01 and ***p < 0.001 compared to the control group; #*p* < 0.05, ##*p* < 0.01 and ###*p* < 0.001 compared to the scopolamine-treated group (*n* = 3).

Acetylcholine (Ach), the first identified neurotransmitter, plays an important role in hippocampal memory function and deeply involved in the pathogenesis of AD (Haam & Yakel, 2017; Jing et al., 2018). choline acetyltransferase (ChAT), the enzyme that synthesizes acetylcholine (Ach), is thought to be present in both cerebrospinal fluid (CSF) and plasma (Karami, Darreh-Shori, Schultzberg, & Eriksdotter, 2021), and acetylcholinesterase (AchE) is an enzyme that metabolizes the Ach at synaptic cleft, resulting in cognitive impairment (Patel, Raghuwanshi, Masood, Acharya, & Jain, 2018). To further evaluate the neuroprotective of GPCRAC extracts on hippocampal neuron damage, cholinergic dysfunction as indicated by decreased Ach content, ChAT activity and increased AchE activity was evaluated. As shown in Figure 2, GPCRAC extracts significantly reversed the decreased content of Ach (Figure 2A), activity of ChAT (Figure 2B), as well as the increased activity of AchE (Figure 2C) in hippocampal tissue of scopolamine-treated mice, especially the high-dose group, confirming an ameliorating effect of GPCRAC extracts on cholinergic function after scopolamine exposure. Together with behavioral experiments, our findings suggested that high-dose GPCRAC extracts were helpful in alleviating scopolamine-induced cognitive impairment, thus, GPCRAC extracts with high dosage was selected for further mechanism studies.

### 3.3 HPLC Analysis Identified Constituents of GPCRAC Extracts

**FIGURE 3:**
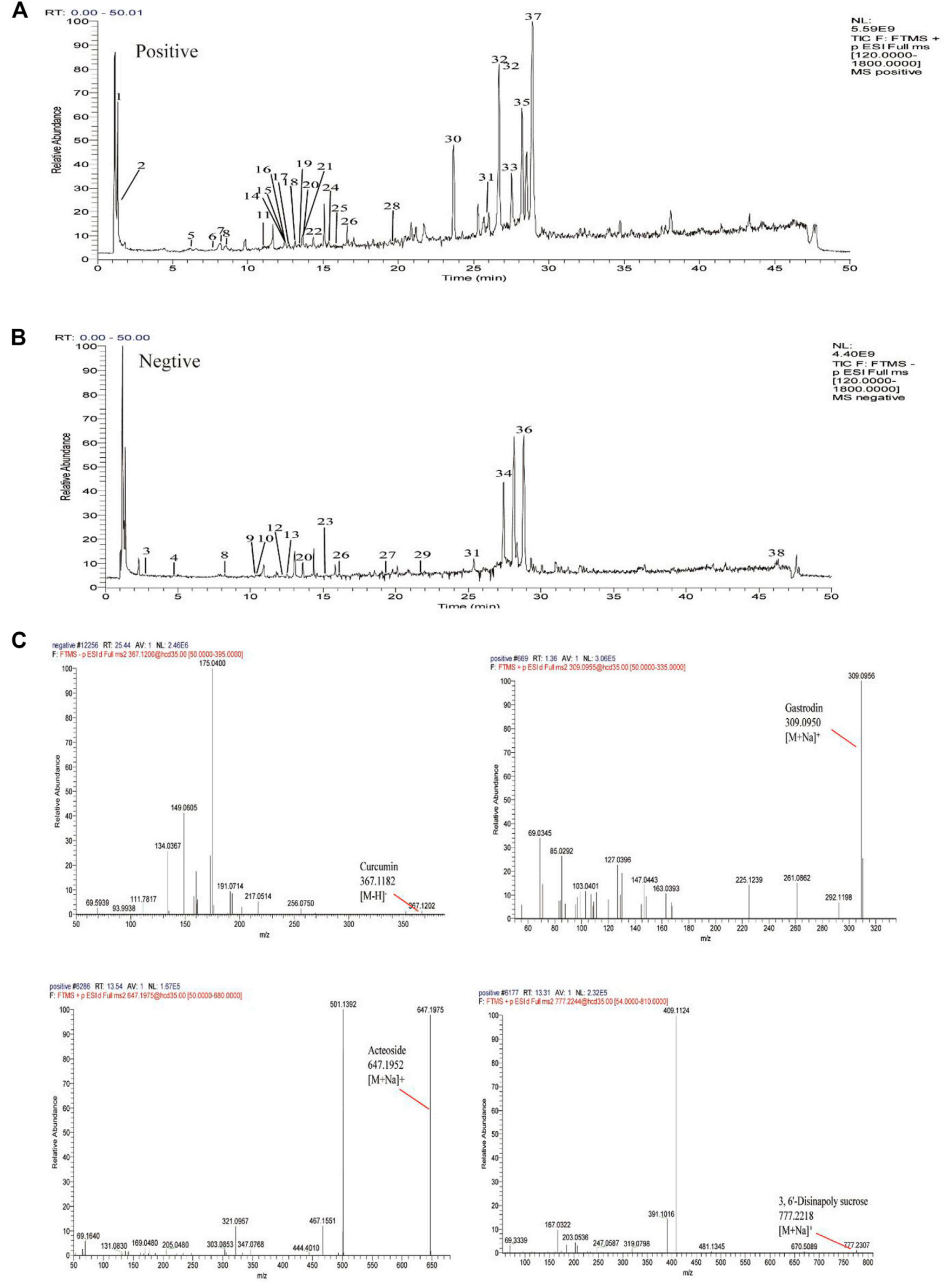
Total ion chromatogram monitored in positive **(A)** and negative **(B)** ion modes for GPCRAC extracts; GPCRAC extracts composition analysis: GPCRAC extracts has Gastrodin, 3, 6′-Disinapoly sucrose, Echinacoside, Acteoside, Curcumin and other ingredients **(C)**.

The mass spectra were recorded across the range of m/z 120-1800. All of the data were processed using Xcalibur software (version 2.7). The major components were well separated and detected under optimized UHPLC and MS conditions (Figure 3). The chemical composition of GPCRAC extracts was determined by comparing the UPLC-Q-Orbitrap MS analysis with the TCMSP database (https://old.tcmspe.com/tcmsp.php) and references. The molecular ion peak in the positive and negative ion mode was employed for the analysis of the relative molecular mass of the compounds. And then the compounds were qualitatively identified by comparing chromatographic retention time and MS/MS information. Finally, 38 compounds were identified from GPCRAC extracts including such as Gastrodin, SibiricoseA5, 3, 6′-Disinapoly sucrose, OnjisaponinB, Echinacoside, Acteoside, Curcumin, 1′, 2′-dihydroxyasarone (Table 1).

**TABLE 1:** | Analysis of the chemical constituents of GPCRAC extracts by UHPLC-Q-Orbitrap in positive and negative ion modes.

Identification	No	RT(min)	Quasi-molecular ion	Measured value	Calculated value	Error(ppm)	Fragment ions	Formula	Sources
Gastrodin	1	1.36	[M + Na] ^+^	309.0956	309.095	1.941	147.0447	C13H8O7	TCMSP
Dihydrocatalpol	2	1.75	[M + Na] ^+^	387.1277	387.1267	2.583	225.0745	C15H24O10	TCMSP
Parishin E or Parishin G	3	2.92	[M-H]^-^	459.1166	459.1139	5.881	397.1151, 173.0089, 161.0450, 129.0187	C19H24O13	Zhang et al. (2021a)
8-Epi-Loganic acid	4	4.93	[M-H]^-^	375.1312	375.1291	5.598	213.0774, 169.0869, 151.0762	C16H24O10	Hu et al. (2021)
Sibiricose A5	5	6.23	[M + Na] ^+^	541.1551	541.1553	−0.37	379.1014, 361.0904, 203.0531, 167.0323	C22H30O14	Zhou and Tu, (2014)
Sibiricose A6	6	7.32	[M + Na] ^+^	571.1659	571.1639	3.502	407.1121, 391.1047	C23H32O15	Zhou and Tu, (2014)
Cistanoside F	7	8.32	[M-H]^-^	487.148	487.1452	5.748	179.0349, 135.0445	C21H28O13	TCMSP
		8.56	[M + Na] ^+^	511.1448	511.1428	3.913	365.0854		TCMSP
Parishin B or Parishin C	8	10.44	[M-H]^-^	727.2122	727.2086	4.95	423.0964, 379.1048, 217.0512, 161.0453, 129.0187	C32H40O19	Zhang et al. (2021b)
Purpureaside C	9	11.05	[M + Na] ^+^	809.251	809.248	3.707	663.1926, 501.1470	C35H46O20	TCMSP
Sibiricaxanthone B	10	12.05	[M-H]^-^	537.1274	537.1244	5.585	243.0309	C24H26O14	Xu et al. (2017)
Parishin A	11	12.41	[M-H]^-^	995.3088	995.3036	5.225	459.1197, 423.0958, 379.1052, 263.0794, 161.0453, 129.0187	C45H56O25	Zhang et al. (2021a)
Cistanoside A	12	12.46	[M + Na] ^+^	823.2662	823.2637	3.037	661.2116, 515.1538	C36H48O20	Li et al. (2015)
Jionoside A1	13	12.46	[M + Na] ^+^	823.2662	823.2637	3.037	677.2081, 515.1538	C36H48O20	Thu et al. (2021)
Polygalaxanthone III	14	12.48	[M + H] ^+^	569.1525	569.1507	3.163	401.0878, 383.0778, 365.0669, 353.0669, 341.0669, 317.0669, 287.0561, 275.0565	C25H28O15	Xu et al. (2017)
Echinacoside	15	12.66	[M + Na] ^+^	809.251	809.248	3.707	647.1993, 501.1407, 483.1497	C35H46O20	Hu et al. (2021)
Tenuifoliside B	16	13.08	[M + Na] ^+^	691.1872	691.185	3.183	409.1123, 391.1016, 323.0751, 167.0322	C30H36O17	Zhou and Tu, (2014)
3, 6′-Disinapoly sucrose	17	13.31	[M + Na] ^+^	777.2244	777.2218	3.345	409.1124, 391.1016, 167.0322	C34H42O19	Zhou and Tu, (2014)
Acteoside or Isoacteoside	18	13.54	[M + Na] ^+^	647.1975	647.1952	3.554	501.1392	C29H36O15	TCMSP
		13.59	[M-H]^-^	623.2009	623.1976	5.295	461.1694, 315.1093		TCMSP
Tubuloside A	19	13.59	[M + Na] ^+^	851.2617	851.2586	3.642	705.2033, 543.1498	C37H48O21	Lei et al. (2021)
2-acetylacteoside	20	15.12	[M-H]^-^	665.2126	665.2082	6.614	503.1795, 461.1695, 443.1577	C31H38O16	Lei et al. (2021)
Tenuifoliside A	21	15.25	[M + Na] ^+^	705.2031	705.2007	3.403	423.1277, 405.1171, 323.0750, 167.0322	C31H38O17	Zhou and Tu, (2014)
Asaronaldehyde	22	15.89	[M + H] ^+^	197.0819	197.0814	2.537	182.0582, 169.0867, 139.0726	C10H12O4	TCMSP
Tubuloside B	23	16.01	[M-H]^-^	665.2126	665.2082	6.614	623.2030, 503.1838, 461.1695, 443.1570, 315.1105	C31H38O16	Han et al. (2012)
		16.8	[M + Na]^+^	689.2079	689.2058	3.047	543.1497		
Desacylsenegasaponin B	24	19.73	[M + Na]^+^	1,289.5828	1,289.5779	3.8	703.3691, 541.3174	C59H94O29	TCMSP
*p*-Hydroxybenzaldehyde	25	21.79	[M-H]^-^	121.0286	121.029	-3.305		C7H6O2	TCMSP
Tenuifolin	26	23.15	[M + Na]^+^	703.3691	703.3669	3.128	673.3590, 541.3170, 511.3088, 493.2847	C36H56O12	Zhou and Tu, (2014)
Onjisaponin R	27	26.03	[M + Na]^+^	1,641.6998	1,641.6937	3.716	1,479.6477, 961.3207	C76H114O37	Liu et al. (2007)
Onjisaponin A	28	26.84	[M + Na]^+^	1727.7356	1727.7305	2.952	1,565.6831, 1,433.6404, 1,155.5414	C80H120O39	Nagai et al. (2001)
Onjisaponin F	29	27.05	[M + Na]^+^	1,611.6893	1,611.6831	3.847	1,449.6301, 769.2548	C75H112O36	TCMSP
Onjisaponin B	30	27.64	[M-H]^-^	1,571.6979	1,571.6906	4.645	177.0556	C75H112O35	Li et al. (2008)
Onjisaponin E	31	28.35	[M + Na]^+^	1,509.6567	1,509.6514	3.511	1,347.6062, 1,069.5078, 703.3694	C71H106O33	Nagai et al. (2001)
Curcumin	32	28.8	[M-H]^-^	367.12	367.1182	4.903	217.0511, 149.0605	C21H20O6	TCMSP
Onjisaponin Y	33	28.82	[M + Na]^+^	1,433.6393	1,433.6354	2.72		C69H102O30	Li et al. (2008)

### 3.4 Quality Assessment of the Proteomic Data

**FIGURE 4:**
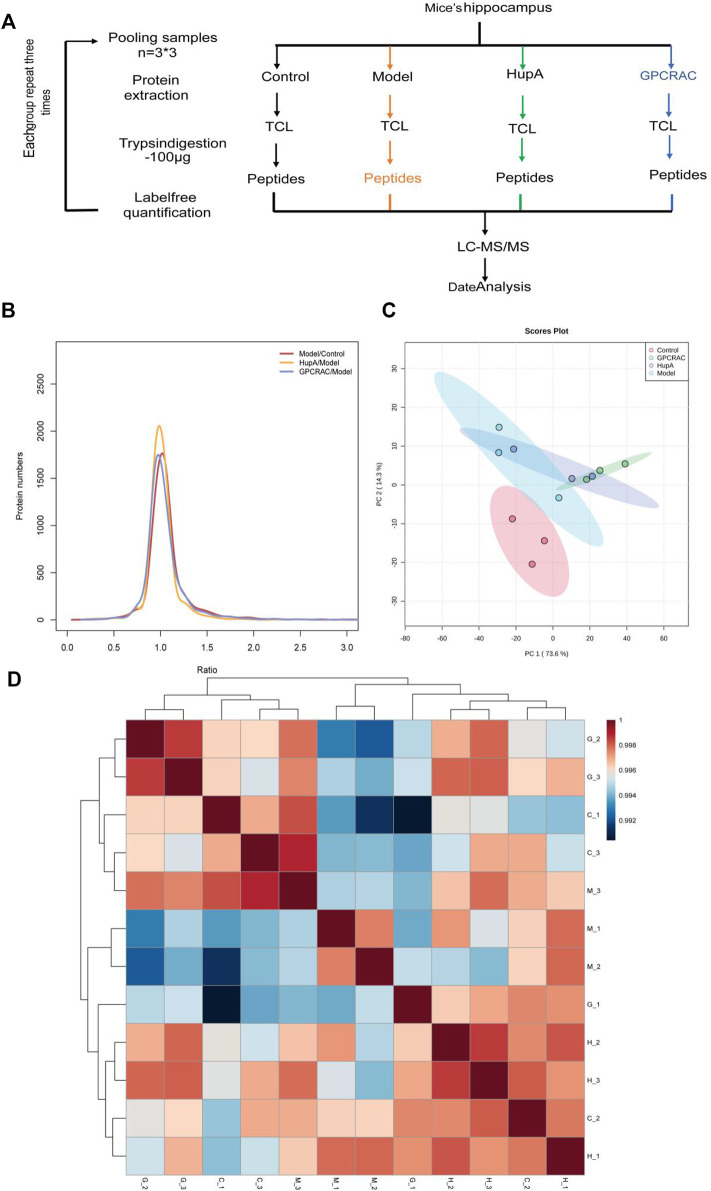
Quality assessment of the proteomic data. Graphical illustration of the workflow used for our LC-MS/MS-based proteomic analysis **(A)**; Log2ratio distribution of all differentially expression proteins detected in each group of three replicate samples, as determined by ANOVA based on FDR <0.05 **(B)**; The PCA analysis of hippocampal proteomes obtained from scopolamine-induced AD mice **(C)**. Sample correlation heat map between different group **(D).**

We performed a high-throughput quantitative proteomic using three replicates of hippocampal proteomes obtained from scopolamine-induced AD mice, the workflow chart based on label-free quantitative proteomics was given in Figure 4A. In detail, to verify whether the hippocampal proteome data of AD mouse model could be applied to further AD studies, we assessed the quantitative reproducibility (differences in technology and experimental conditions) of the hippocampal proteomes. Normalized protein expression of per sample revealed similarly distributions (Figure 4B). Moreover, principal component analysis (PCA) was applied to characterize the degree of proteome alterations within and between hippocampal samples. As demonstrated in Figure 4C, the first two principal components (PC1 and PC2) account for 87.9% of the overall cumulative variance contribution rate. It also showed a clear distinction between the control and model groups. Furthermore, as compared to the model group, the varied samples from the GPCRAC extracts group were more compactly packed, demonstrating superior reproducibility. Similarly, as shown in Figure 4D, we discovered strong Pearson correlations among biological replicates of the same groups (R, 0.990–1) and significantly lower correlations across different groups. The distribution of samples correlation heatmap also indicated a good biological reproducibility of the dataset. In conclusion, the observed variations in quantitative protein expression reflect the variety of molecular alterations across groups in mouse tissues rather than the effect of our label-free strategy. Thus, the workflow for proteomic analysis was stable and reliable, and the high degree of quantitative accuracy offered by the label-free quantification (LFQ) technique allows us to investigate proteomic alteration in the pathological development of AD.

### 3.5 Analysis of Differentially Expressed Proteins

**FIGURE 5:**
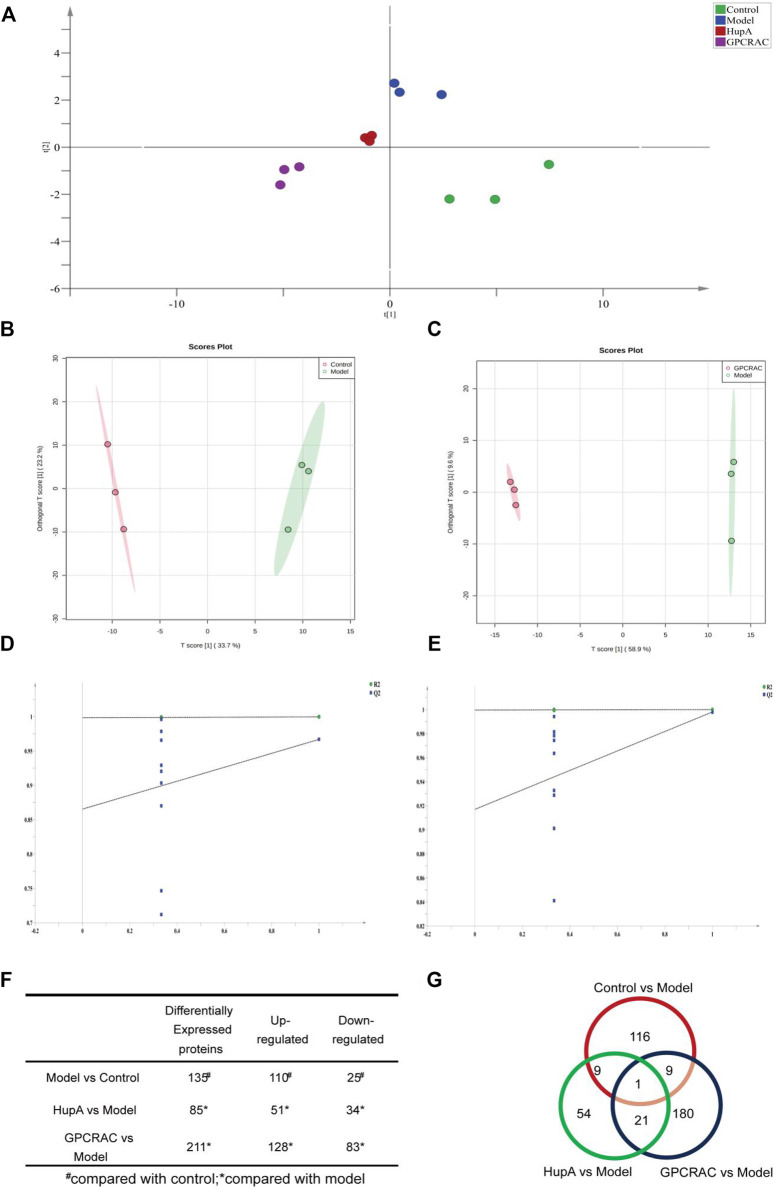
Evaluation of differentially expressed proteins; PLS-DA scores plot of different group samples **(A)**; OPLS-DA scores plot model revealed a clear separation between control vs. model **(B)** and model vs. GPCRAC extracts **(C)** groups; The corresponding permutation test **(D,E)**; The fold-change cutoff was set at ≥ 1.2 or ≤0.883 with *p* < 0.05. Identified up- or down-regulated proteins in hippocampus proteomics from each group **(F)**; Venn diagram analysis of differentially expressed proteins **(G)**.

To characterize the differentially expressed in protein profiles, the Partial Least-Squares Discriminant Analysis (PLS-DA) model was used to further optimize the population separation of different groups (Figure 5A). Orthogonal Partial Least Squares-Discriminant Analysis (OPLS-DA) analysis, a supervised identification method, was applied to strengthen an established separation between the control vs. model group (Figure 5B, R2Y = 0.941, Q2 = 0.685) and model vs. GPCRAC group (Figure 5C, R2Y = 0.986, Q2 = 0.79). Subsequently, the permutation test was used to evaluate the possible overfitting of the OPLS-DA model. The corresponding validation plot showed that all of the PLS-DA models built for duplicate samples at different groups were valid (Figures 5D,E). Results obtained by OPLS-DA classification reveal a clear segregation of each group, indicating the significant changes in the expression of differentially expressed proteins in different groups. The proteins with $\frac{MC}{NC}$ fold-change>1.2 or <0.83 and *p* < 0.05 were chosen for further analysis. A subset of 4,112 proteins were quantified (unique peptides ≥ 2), and 390 quantified proteins were identified as differential expression proteins. Changes in protein levels were shown in Figure 5F, specifically, there are 135 differentially expressed proteins between control and model group, of which 110 are significantly up-regulated proteins and 25 are significantly down-regulated proteins. And, we identified 211 proteins regulated by GPCRAC extracts (128 up-regulated and 83 down-regulated). The overlaps of differentially expressed proteins between model vs. control and GPCRAC extracts vs. mod were 9 (Figure 5G).

### 3.6 The Integrated Bioinformatics Analysis Indicated the Potential Targets Mediated by GPCRAC Extracts in Treating AD

The integrated bioinformatics analysis based on proteomics was undertaken to discover the hub target proteins mediated by GPCRAC extracts in treating AD.

**FIGURE 6:**
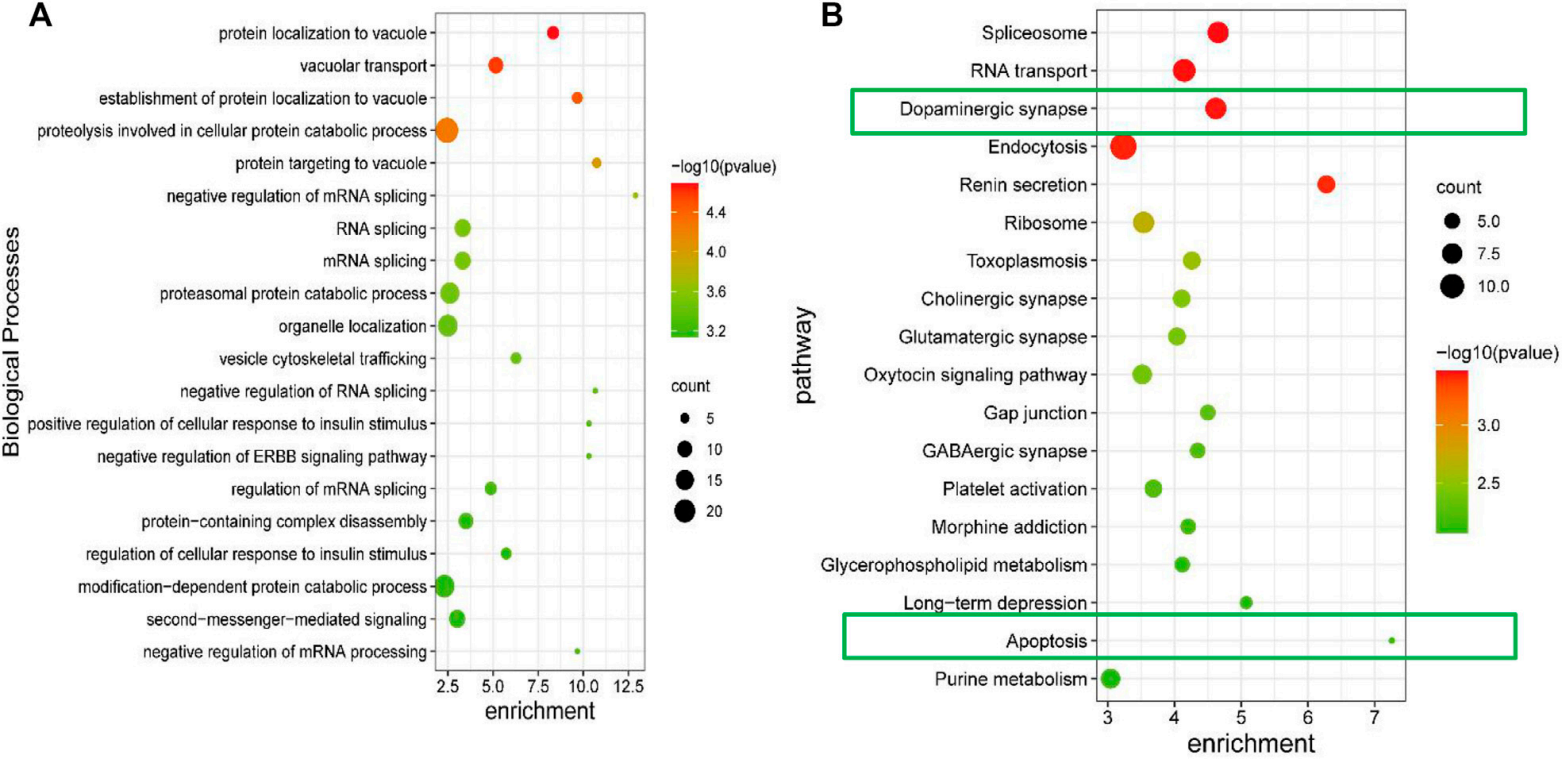
GO enrichment and KEGG pathway analysis of differential expressed proteins. The biological process of differentially expressed proteins derived from the GO analysis **(A)**; KEGG signaling pathway analysis for the differentially expressed proteins, the *p*-value was set at <0.05 **(B)**.

To begin, the GO and KEGG analysis were perform to explore the potential functions of the differentially expressed protein involved in the GPCRAC extracts-mediated treatment of AD. The biological process of Gene Ontology (GO) analysis revealed that these differentially expressed proteins were primarily enriched in the positive regulation of autophagosome, presynaptic active zone membrane, ATPase activator activity, postsynaptic potential, postsynapse assembly, which is consistent with the fact that the pathology of AD correlates positively with synaptic function in hippocampal neurons (Figure 6A). KEGG analysis showed relevant pathways with *p* < 0.05 (Figure 6B). Spliceosome, RNA transport, Ribosome, Dopaminergic synapse, Long-term depression, Glutamatergic synapse, GABAergic synapse, Apoptosis, and other highly enriched terms were found in the KEGG pathway analysis. We discovered that most of the pathways play an important role in modulating the neurological function of the hippocampal, with the dopaminergic synaptic pathway being the best discriminant neural-related pathway, indicating that the dopaminergic pathway may be a necessary signaling pathway of target enrichment for the treatment of AD. Furthermore, neuronal apoptosis is a well-known pathway implicated in the degenerative process of AD.

**FIGURE 7:**
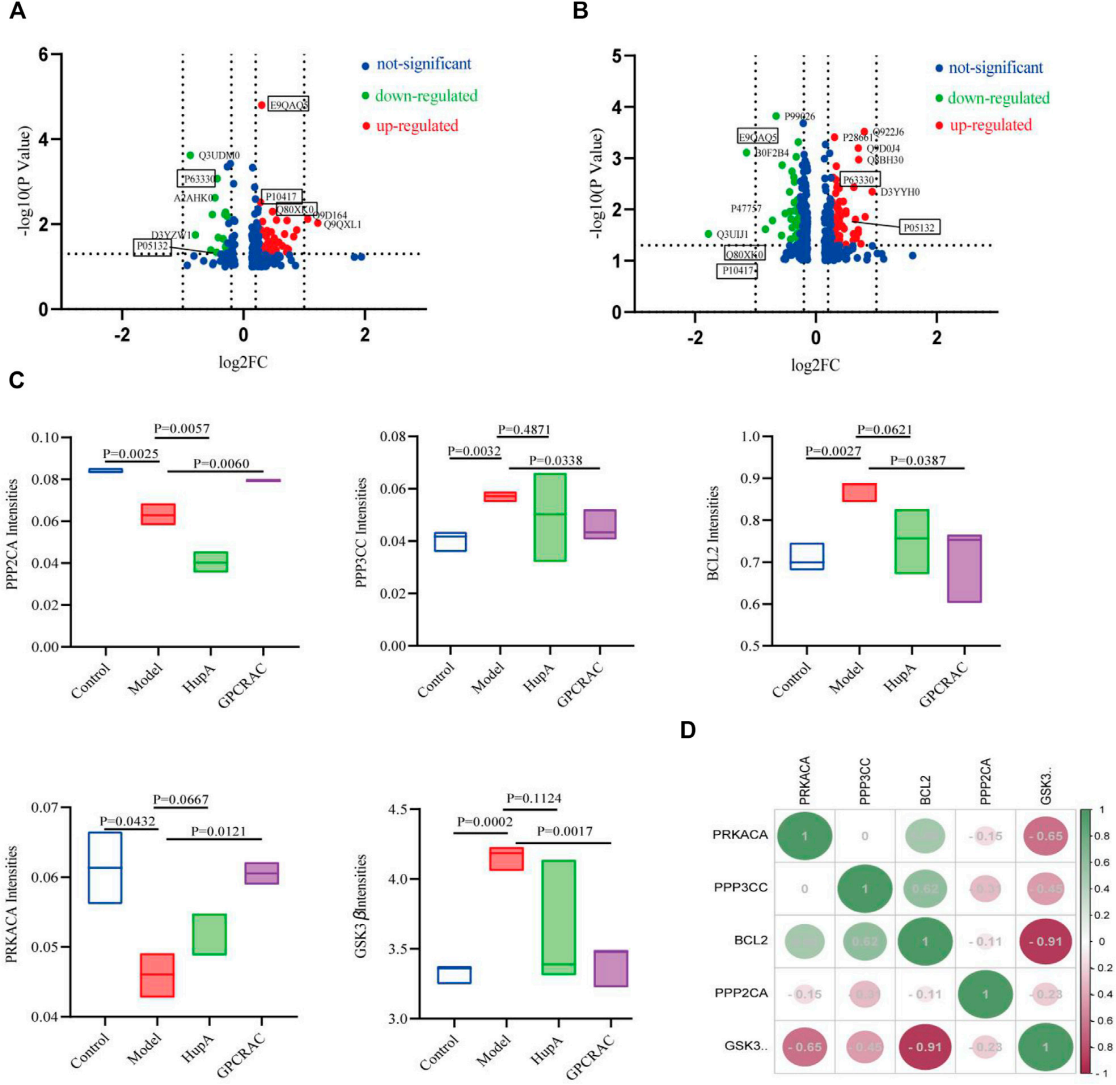
Mapping of hub significantly regulated proteins. Volcano plots show the distribution of the significantly altered proteins between control vs. model **(A)** and model vs. GPCRAC extracts **(B)** groups; Five proteins (PPP2CA, GSK3β, PPP3CC, PRKACA, and BCL-2) were identified as hub differential expressed protein in hippocampus of AD mice. The intensity of protein is given as the normalized protein abundance. Significant change levels of five proteins were presented in the box plots **(C)**; Correlation coefficients heatmap of hub differentially expressed protein **(D)**.

Next, to identify the core protein targets mediate by GPCRAC extracts in treating AD, volcano plot mapping was constructed to detect the proteins that were significantly regulated between control vs. Model (Figure 7A) and model vs. GPCRAC extracts (Figure 7B) groups. Specifically, PPP2CA (p63330), PRKACA (P05132), PPP3CC (Q80XK0), GSK3β (E9QAQ5) and BCL-2 (P10417) were confirmed a clearly altered protein profiles in model vs. control group, as well as these proteins were dramatically reversed following GPCRAC extract therapy**.** To examine the specific change levels of these significantly altered proteins that were genetically associated to AD, the ‘protein expression level’ represented by the ratio of individual samples compared to pooled samples (called “normalized protein abundance”) was investigated**.** As shown in Figure 7C, the expression level of GSK3β, PPP2CA, PRKACA, PPP3CC, and BCL-2, consist with volcano plot maps, were recovered after the administration of the GPCRAC extracts. Of these hub proteins, PPP2CA and PRKACA were down-regulated and GSK3β, PPP3CC and BCL-2 were down-regulated in AD group. Following that, we exploit Pearson’s correlation heatmap to interpret the ‘signs’of interactions (ie, activation-inhibition relationships) between core differential proteins. The correlation matrix data (Figure 7D) showed positive or negative correlations between these core regulated proteins, with PPP3CC and PRKACA being positively linked with BCL-2 and PPP2CA and GSK3β being negatively correlated.

**FIGURE 8:**
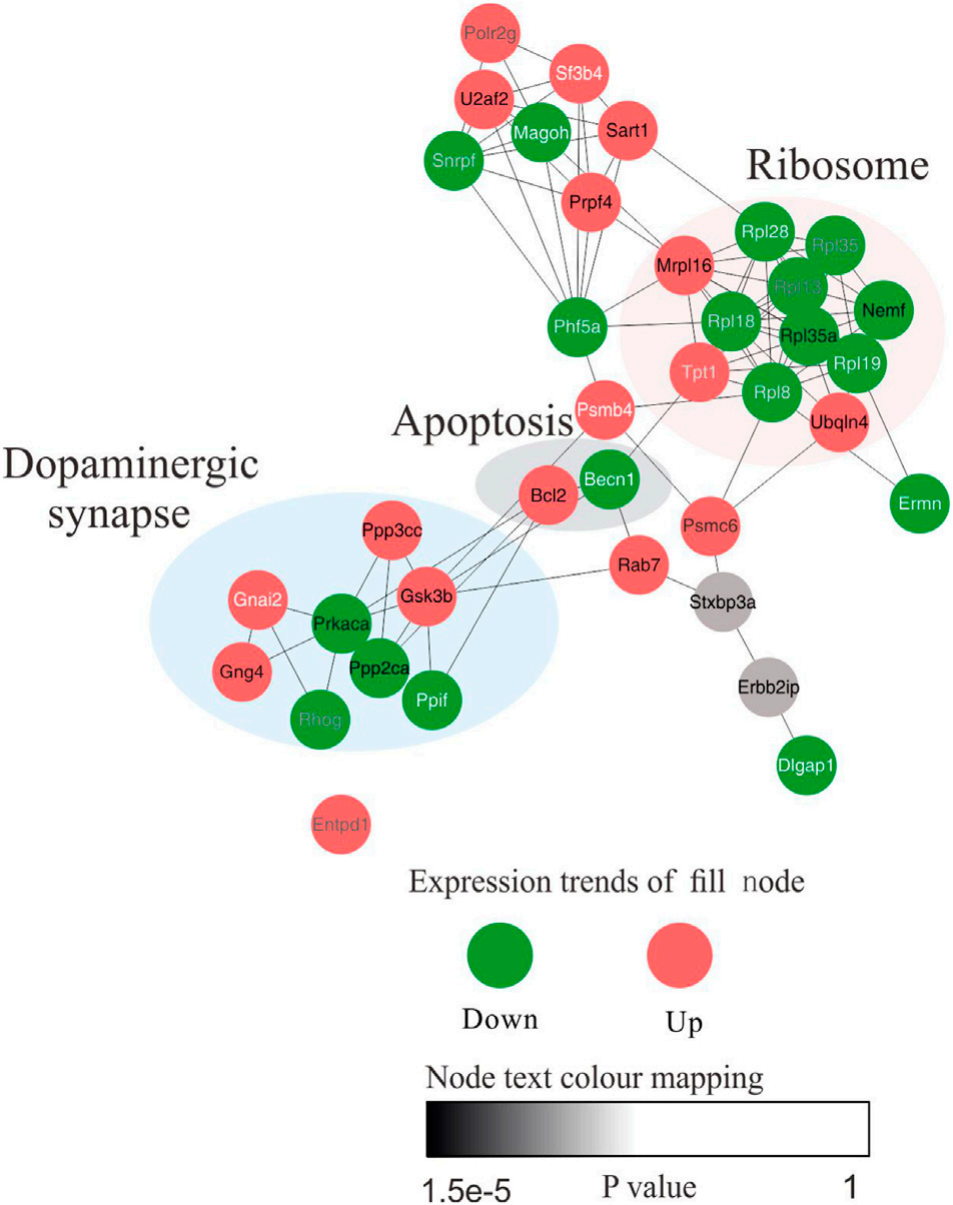
The network model was constructed to elucidate the biological interpretations of hub proteins in the core network. Functional protein–protein interaction (PPI) network of the 135 proteins between control and model group was constructed. Top 35 nodes ranked by MCC. The node fill colour bar represent up-regulated (red) or down-regulated (green). The node text colour bar represents the *p*-value.

We next sought to further attest the biological interpretations of hub proteins in the core network. Briefly, protein–protein interaction (PPI) networks with Cytohubba plugins of Cytoscape was conducted to characterize hub proteins flow within core networks. The top 35 nodes were first ranked using Maximal Clique Centrality (MCC) method. The pivot protein and its biological function were eventually discovered. PPI visualization result suggest that several of the altered proteins were significantly connected with each other (enrichment value *p* < 0.005) and most of the hub differential proteins were primarily concentrated in the dopaminergic synapse and apoptosis signaling pathways, moreover, these pathways and some identified core proteins are implicated in AD pathogenesis (Figure 8). Eventually, PP2CA, PRKACA, PPP3CC, GSK3β associated with dopaminergic synapse and BCL-2 were selected as further analysis targets due to their closely relevance to neuronal damage.

### 3.7 Validation of the Protein Targets

**FIGURE 9:**
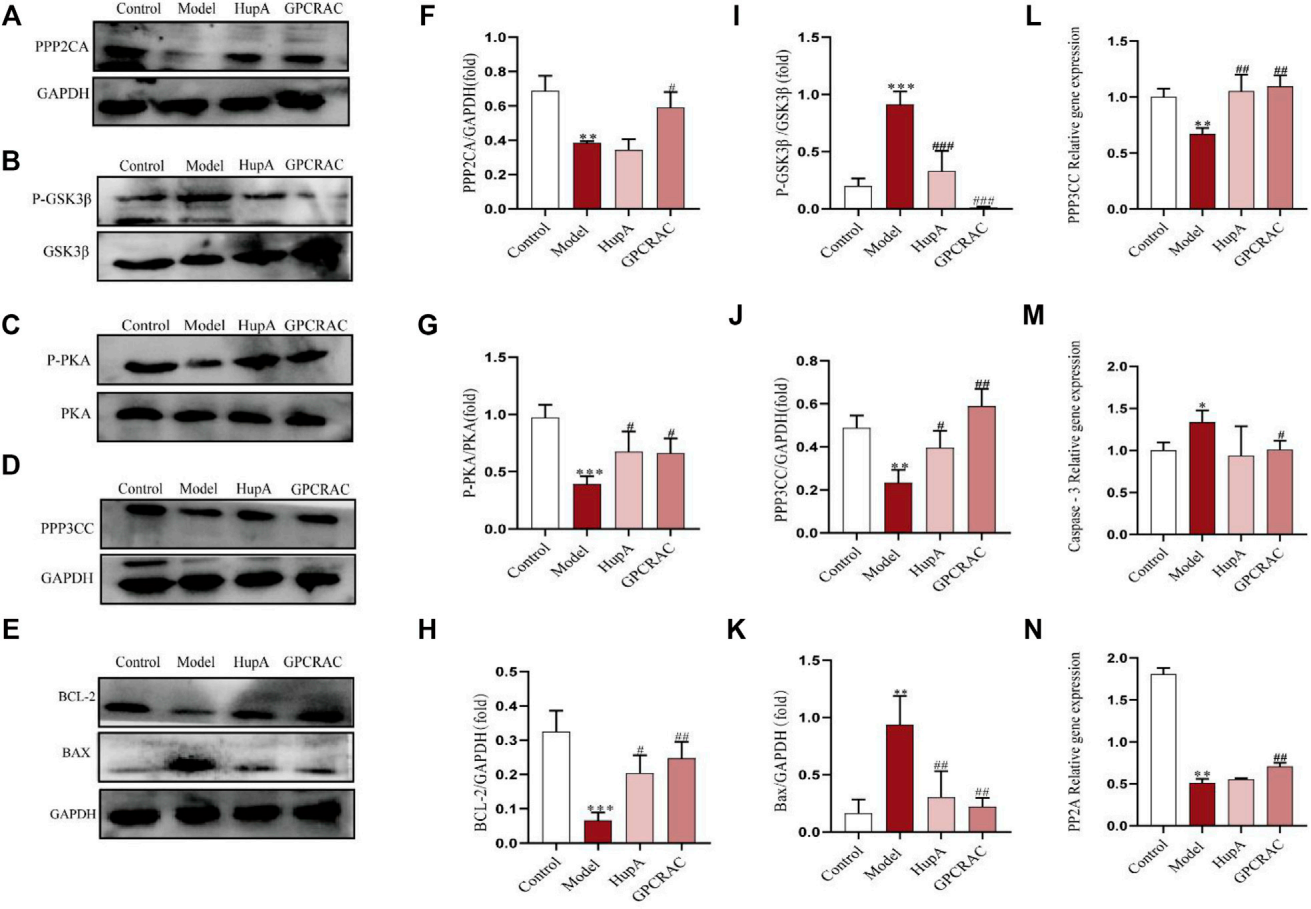
RT-qPCR and WB verified the significantly changed proteins. Representative images **(A–E)** and the protein levels of PPP2CA, GSK3β, PRKACA, PPP3CC, BCL-2, Bax in hippocampus tissues from mice were measured by Western blotting respectively (*n* = 3) **(F–K)**; Relative mRNA expression of PPP2CA, PPP3CC and Caspase-3 in hippocampus tissues were detected by real time-PCR analysis respectively (*n* = 3) **(L–N)**; Data were expressed as mean ± SE, *p < 0.05, **p < 0.01 and ***p < 0.001 compared to the control group; #*p* < 0.05, ##*p* < 0.01 and ###*p* < 0.001 compared to the scopolamine-treated group (*n* = 3).

The hub differentially expressed proteins and pathways of GPCRAC extracts in mediating AD were further verified by western blot and qPCR. PPP2CA encoding the alpha (*α*)-isoform of catalytic subunit of serine/threonine-protein phosphatase 2A (PP2A) (Bhardwaj et al., 2014). PP2A and GSK3β are essential proteins involved in the regulation of synaptic plasticity in the dopaminergic synaptic pathway (Martin, Magnaudeix, Wilson, Yardin, & Terro, 2011), and they are linked to aberrant tau phosphorylation and neuronal death in AD (Wang et al., 2019). Additionally, PPP3CC and PRKACA are also important regulators of synaptic and neural plasticity, Figure 9 illustrates this. Representative western blotting images (Figures 9A–E) and fold changes in relative densitometric values of PPP2CA, P-PKA, PPP3CC, P-GSK3β, BCL-2 and Bax were determined by western blotting. In the hippocampus of mice exposed to scopolamine, reduced expression of PPP2CA, P-PKA, PPP3CC (Figures 9F,G,J) and over-activated P-GSK3β expression (Figure 9I) (*p* = 0.005) was detected as compared to controls. Intriguingly, high dosages of GPCRAC extracts caused a considerable reversal in the expression levels of PPP2CA, PPP3CC, P-PKA, and P-GSK3β. We also found lower levels of PPP2CA and PPP3CCmRNA expression in the scopolamine-treated model mice relative to the control animals (Figures 9L,N), which was restored by treatment with GPCRAC extracts.

The apoptosis signaling system is important pathway in animal growth and tissue homeostasis (Cheng & Ferrell, 2018). We discovered apparent alterations in the apoptosis-related protein BCL-2 using proteomics, therefore we wanted to learn more about the level of apoptosis in the hippocampus of mice given by scopolamine and the protective effects of GPCRAC extracts. Furthermore, qPCR and Western blotting were used to investigate the anti-apoptotic efficacy of GPCRAC extracts in the hippocampus of mice subjected to scopolamine. When compared with control animals, obviously higher protein expression levels of Bax (Figure 9K) and gene expressions levels of Caspase-3 (Figure 9M) were discovered, as well as lower protein levels of BCL-2 (Figure 9H). In the mouse brain, treatment with GPCRAC extracts drastically reduced the expression of Bax and Caspase-3 while increasing the expression of BCL-2. These findings shown that GPCRAC extracts may dramatically regulate the expression of dopaminergic synapse associated proteins and apoptosis-related proteins in scopolamine-induced mice brain tissues, thereby treating AD.

## 4 Discussion

The use of TCM as a potential therapeutic treatment for AD is an evolving field of investigation. However, there is currently a lack of robust experimental proof and mechanism elaboration of TCM treatment. A more effective therapy is being researched. In this work, we first investigated the neuroprotective impact of GPCRAC extracts in reducing cognitive impairment evoked by scopolamine. Scopolamine, a muscarinic cholinergic receptor antagonist, is frequently employed as a typical pharmacological model for producing cognitive impairment in animals (Shabani & Mirshekar, 2018). According to reports, scopolamine activity is predominantly associated to cholinergic depletion and apoptosis in the brain, both of which are hallmarks of the disease (Sohn, Lim, Kim, Kim, & Jeong, 2019; Chen & Yeong, 2020). Based on the scopolamine-induced cognitive impairment, we studied the behavioral phenotype of AD model mice and discovered that GPCRAC extracts at high doses markedly alleviate AD-related cholinergic dysfunction, as demonstrated by increased Ach content and ChAT activity and decreased AchE activity. Furthermore, behavioral tests revealed that scopolamine-triggered mice’s hippocampus showed cognitive dysfunction in spatial learning and memory abilities. Apart from the study of protective efficacy of GPCRAC extracts in improving cognitive impairment, acute oral toxicity and 13 weeks sub-acute toxicity studies were also conducted previously. Safety evaluations on clinical observation, body weight, hematology, histopathological examination, ophthalmological examination did not show any adverse changes. Together with previous work, the current study proven that the administration of GPCRAC extracts is both efficacious and safe.

Currently, even though several signaling pathways have emerged as a remarkably effector for AD treatment, there is still under investigating of the mediation of TCM to potential signaling pathway during AD pathological process. The natural herbs are usually more difficult to be clarified in a specific pathway due to its’ complex components especially for compound. For further understand the mechanism of action of GPCRAC extracts alleviating AD, we employed in-depth quantitative proteomics to characterize the abnormal alterations of the hippocampal proteomes obtained from scopolamine-induced AD mice. Our MS-based strategy identified 390 proteins that were shared by different groups. By mapping the significantly regulated proteins, we identified five hub proteins: PPP2CA, GSK3β, PPP3CC, PRKACA, BCL-2. Surprisingly, the proteins that were significantly altered were heavily enriched in dopaminergic synaptic signaling and apoptosis signaling pathway, respectively. It is therefore possible that these hub proteins and related pathways may serve as possible mechanisms for the treatment of AD.

**FIGURE 10:**
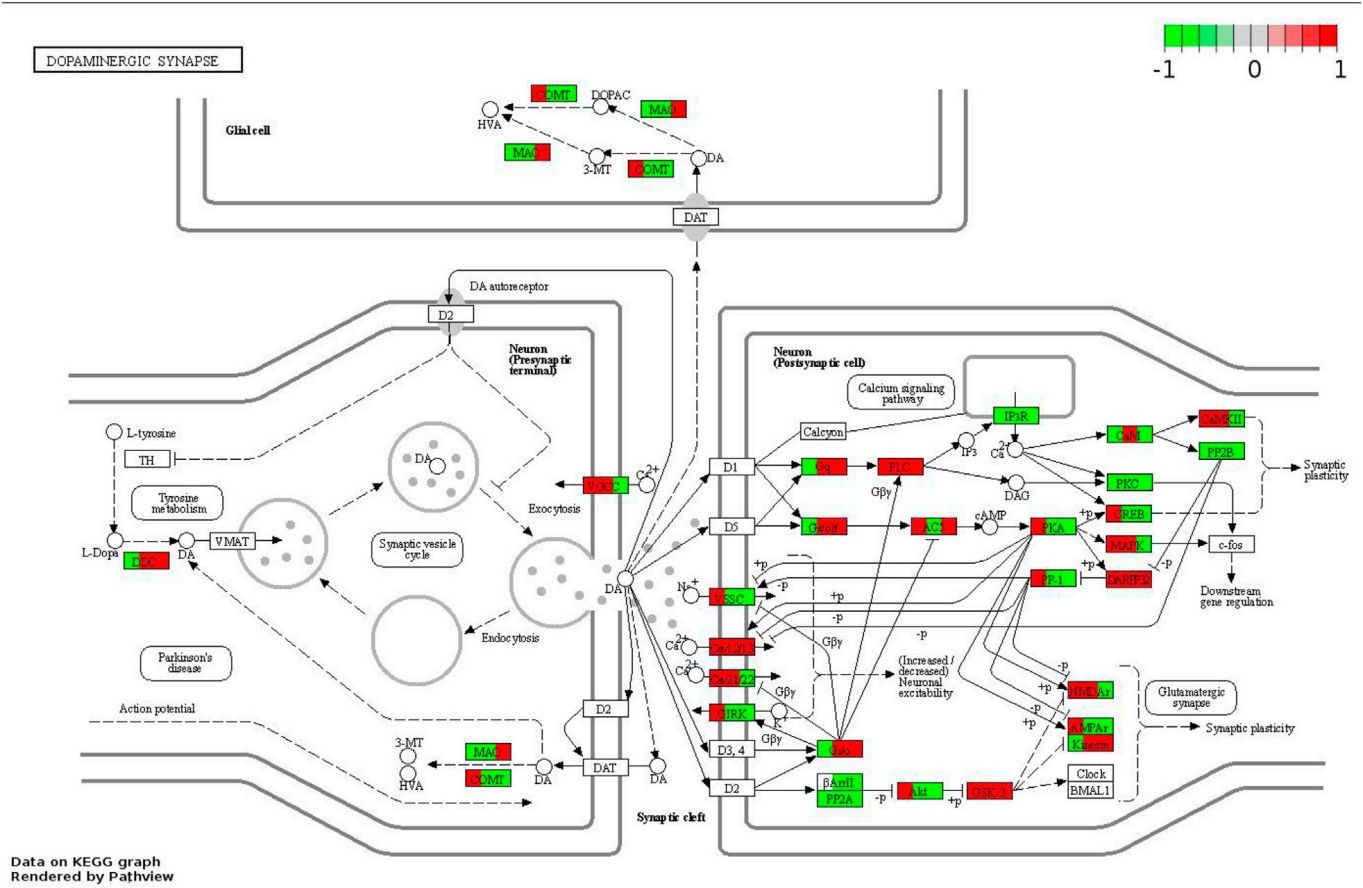
Modulating the dopaminergic synapse signaling pathway of GPCRAC extracts against AD. Core differentially expressed protein mediated by GPCRAC extracts are colored in red (up-regulated) and green (down-regulated).

As one of the important signal transduction pathways in AD, the dopaminergic synapse signaling pathway plays an important biological role in the process of neurotransmitter coordination, memory consolidation, neuronal synapse function (Bamford, Wightman, & Sulzer, 2018). Protein phosphatase 2A (PP2A), found at higher concentrations in brain, is a crucial phosphatase (Reynhout et al., 2019). And accept that the PP2A upregulation has been linked to a reduction in tau hyperphosphorylation and amyloid formation (Liu et al., 2013; Liu et al., 2016). Recently evidence manifest that PP2A-mediated protein dephosphorylation is important for the homeostasis of synaptic plasticity (Biswas, Cary, & Nolta, 2020). The GSK3β is a kinase that plays a role in the etiology of AD, and it performs biological functions after being dephosphorylated (p-GSK3β) by upstream proteins like PP2A (Elgendy et al., 2019). Also, GSK3β may be explored as a potential therapeutic target for AD through a mechanism that improves dopamine-dependent memory behaviors via modulating D2 receptor (D2R)-mediated signaling (Li et al., 2020), supporting the association to dopaminergic synapse pathway. Furthermore, the interaction between GSK3β and PP2A have been established, that PP2A, the main phosphatase could regulate GSK3β activity by removing phosphorylation at serine 9 (Elgendy et al., 2019). Our proteomics research further adds to the evidence for a negative relationship between PP2A and GSK3β, which is critical for both basal synaptic transmission and long-term memory function. In line with the results of proteomics, our *in vivo* experiments confirmed decreased expression of PP2A and increased phosphorylated GSK3β (p- GSK3β) in the hippocampus of the model group, which further supports the hypothesis that the balance of PP2A and GSK3β, which coordinate synaptic plasticity function, may be an important molecular mechanism in AD treatment. Additionally, the cAMP-mediated activation of protein kinase A (PKA, corresponds to the gene symbol PRKACA), a classical downstream target of dopaminergic synaptic pathway, play a vital role during the activation of presynaptic long-term plasticity (Kaeser et al., 2008; Zhang, Shen, Wu, Zhang, & Xing, 2020). Phosphorylated PKA (P-PKA) may be a key target for GPCRAC extracts in the treatment of AD, according to our findings from proteomics and western blot analyses. In addition, the serine/threonine-protein phosphatase (PPP3CC), which encodes a catalytic subunit of calcineurin (CaN; also named protein phosphatase 2B, PP2B) (Cottrell et al., 2016), was also a novel indicator involved in the differential expressed proteins we screened. It was proved that calcineurin could regulate glutamatergic transmission by indirectly influencing NMDARs that is crucial for neuronal communication (Lin, Sun, Kung, Dell'Acqua, & Hoffman, 2011), and the observed increase in NMDAR might be attributable to the increased levels of PPP3CC (Ambrozkiewicz et al., 2020). According to our proteomics findings, PPP3CC protein expression was up-regulated in the model group. However, utilizing Western blotting and qPCR verification, a drop in PPP3CC protein and gene expression levels was found in the AD mice hippocampus, which was comparable to the earlier findings by Eastwood et al. They also discovered lower levels of PPP3CCmRNA expression in hippocampal neurons under pathological conditions *in vivo*, suggesting that such reductions in expression may contribute to synaptic plasticity failure (Eastwood, Burnet, & Harrison, 2005). Notably, our finding adds to the evidence that PPP3CC level decreases with the pathological progression of AD, and highlights the important role of PPP3CC that may present as a novel protein biomarker for AD. However, the role of PPP3CC in AD has not been fully described previously, its mechanism to prevent nerve damage needs further investigation. Overall, GPCRAC extracts may alleviate the cognitive impairment by regulation of the dopaminergic synapse signaling pathway (Figure 10).

In addition to the dopaminergic synaptic pathway related proteins, the apoptosis pathway related protein BCL-2 shown significantly altered profiles. Dopamine deficiency-mediated nerve cell apoptosis has been shown in studies to be an important pathological basis of neurodegenerative diseases (Zhang et al., 2019), and it has been proposed that neuronal apoptosis caused by cholinergic deficit may be one of the main causes of scopolamine-induced cognitive decline (Sohn et al., 2019). Cell survival or death cascades can be initiated by increasing the anti-apoptotic protein BCL-2 (Delbridge & Strasser, 2015). The variation data shown here reveal that BCL-2 expression was considerably up-regulated in the scopolamine-treated group, which contradicts the results of the western blot. This contradictory finding might be attribute to sample and processing differences. Furthermore, Bax and capease3 concentrations also had large positive differences following GPCRAC extracts. Taken together, these results support the conclusion that the neuroprotective effect of GPCRAC extracts treatment against AD may be through modulation of dopaminergic synapse cascade and apoptosis pathway extrapolated from bioinformatics analysis. Collectively, our work provided novel insights into the molecular mechanism of synaptic plasticity alterations in the pathological process of AD, and further confirmed the effectiveness of GPCRAC extracts as a natural herbal formula against progressive AD. However, the detailed molecular mechanism mediated by GPCRAC extracts is primarily unknown and deserves further investigation. In conclusion, the results of our study demonstrate that GPCRAC extracts treatment can effectively alleviate scopolamine-induced cognitive impairment. The underlying mechanism may be related to the regulation of the dopaminergic synapse/apoptosis pathway.

## Data Availability

The datasets presented in this study can be found in online repositories. The names of the repository/repositories and accession number(s) can be found below: https://www.iprox.cn/page/HMV006.html, ProteomeXchange ID: PXD029640.
