# Supplementary material for: Chinese Herbal Extracts Exert Neuroprotective Effect in Alzheimer’s Disease Mouse Through the Dopaminergic Synapse/Apoptosis Signaling Pathway
Source: Front Pharmacol. 2022 Feb 28;13:817213. doi: 10.3389/fphar.2022.817213 (PMC8918930; doi:10.3389/fphar.2022.817213)
Supplement: Supplementary file 1 [file DataSheet3.ZIP › original images of WB.pdf]

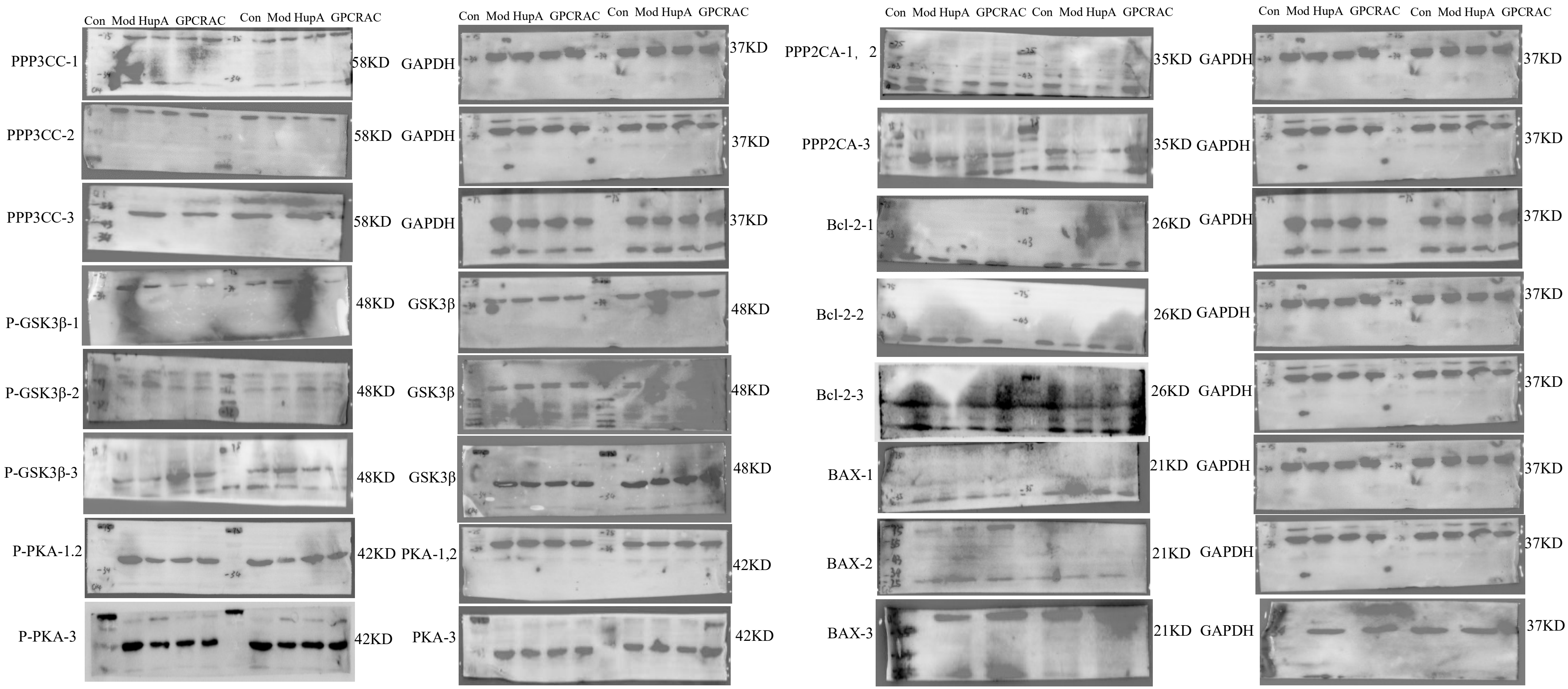

Figure S1: The images for Western blot represent the expression level of PPP2CA, P-Gsk3β, Gsk3β, PP3CC,P-PKA, PKA, BCL2,BAX, Capease3 and the loading control GAPDH
