# Supplementary material for: Chinese Herbal Extracts Exert Neuroprotective Effect in Alzheimer’s Disease Mouse Through the Dopaminergic Synapse/Apoptosis Signaling Pathway
Source: Front Pharmacol. 2022 Feb 28;13:817213. doi: 10.3389/fphar.2022.817213 (PMC8918930; doi:10.3389/fphar.2022.817213)

Fingerprint method of *Acorus gramineus Aiton* extract

1. Apparatus and equipment

High Performance Liquid Chromatograph (HPLC) Shimadzu LC-20A

Electronic balance 1/100,000 analytical balance (Mettler Toledo MS105DU)

2. Reagents and materials

Acetonitrile (Fisher chromatographic purity), water (Watsons distilled water); microporous filter membrane (BOJIN nylon 0.22 μm), syringe (1 mL of Jiangxi Qingshantang Medical Equipment)

3. Reference chromatographic conditions

Shimadzu InertSustain AQ-C18 (4.6×250 mm, 5 μm)

4. Chromatographic conditions and system adaptability test

Use octadecylsilane-bonded silica gel as filler; use acetonitrile as mobile phase A and distilled water solution as mobile phase B, and perform gradient elution as specified in the following table; detection wavelengths are 220 nm and 320 nm.

Times（minutes）	Mobile phase A (%)	Mobile phase B (%)
0.00～5.00	5	95
5.00～8.00	5→8	95→92
8.00～11.00	8	92
11.00～17.00	8→13	92→87

17.00～23.00	13	87
23.00～26.00	13→17	87→83
26.00～30.00	17	83
30.00～31.00	17→21.5	83→78.5
31.00～73.00	21.5→31	78.5→69
73.00～93.00	31→50	69→50
93.00～103.00	50→60	50→40
103.00～108	60→70	40→30
108.00～110.00	70→100	30→0
110.00～120.00	100	0

Reference substance map

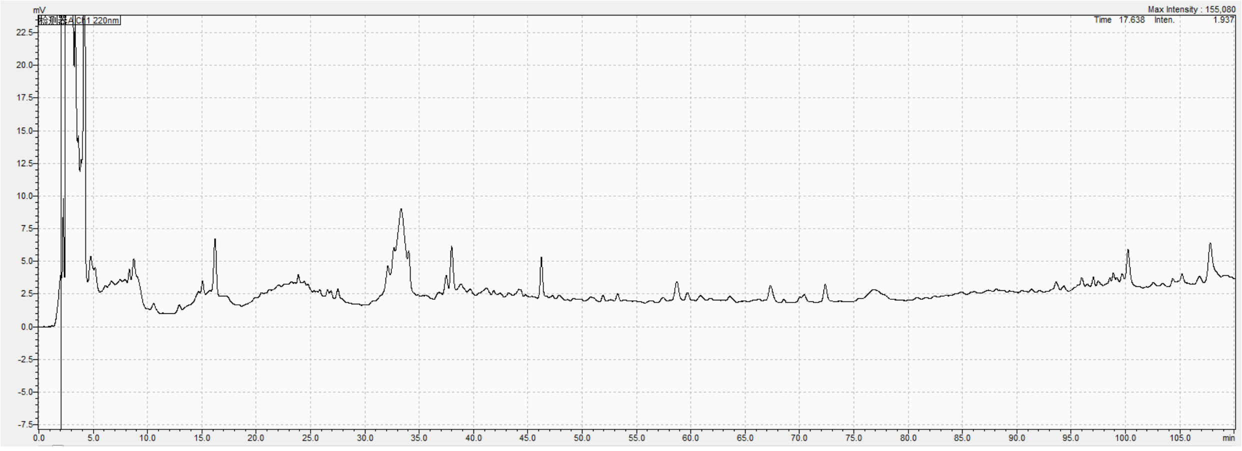

Supplement: Supplementary file 3 [file DataSheet2.ZIP › Acorus gramineus Aiton extracts.pdf]
