# Supplementary material for: Chinese Herbal Extracts Exert Neuroprotective Effect in Alzheimer’s Disease Mouse Through the Dopaminergic Synapse/Apoptosis Signaling Pathway
Source: Front Pharmacol. 2022 Feb 28;13:817213. doi: 10.3389/fphar.2022.817213 (PMC8918930; doi:10.3389/fphar.2022.817213)

1.Measurement of the content of Phenyl Ethanol Glycosides in *Cistanche deserticola Ma*

1.1 Apparatus and equipment

High performance liquid chromatograph (HPLC) (Shimadzu LC-20A); 1/100,000 analytical balance (Mettler Toledo MS105DU)

1.2 Reagents and materials

Methanol (Fisher chromatographic purity), formic acid (Chemical reagent analysis grade of Sinopharm Group), water (Watsons distilled water); Echinacoside reference substance (China Food and Drug Control Institute 20 mg), Acteoside reference substance (China Food and Drug Control Institute 20 mg); microporous filter membrane (BOJIN nylon 0.22 μm), syringe (1 mL of Jiangxi Qingshantang Medical Equipment)

1.3 Reference chromatographic conditions

Shimadzu InertSustain AQ-C18 (4.6×250 mm, 5 μm)

1.4 Chromatographic conditions and system adaptability test

Use octadecyl silane-bonded silica gel as filler; use methanol as mobile phase A and 0.1% formic acid aqueous solution as mobile phase B, and perform gradient elution as specified in the following table; detection wavelength is 330 nm. The number of theoretical plates should not be less than 3000 based on echinacoside peak.

Time (minutes)	Mobile phase A (%)	Mobile phase B (%)
0-17	26.5	73.5

17-20	26.5-29.5	73.5-70.5
20-27	29.5	70.5
27-37	29.5	70.5

1.5 Preparation of reference solution

Weigh echinacoside and Acteoside reference substance, add 10% acetonitrile to make a solution containing 0.20mg per 1mL, as the reference solution.

1.6 Preparation of test solution

Accurately weigh an appropriate amount of the sample (the concentration of echinacoside in the sample solution is equivalent to that of the reference substance) in a 50 mL measuring flask, add about 40 mL of 10% acetonitrile, sonicate for about 30 minutes, take it out and let it cool to room temperature, and dilute with 10% acetonitrile to Grad, shake well, filter with 0.22μm filter membrane to get.

1.7 Determination method

Precisely draw 10 μL each of the reference solution and the sample solution, and separately inject the samples for determination.

Calculation formula:

Where A1: the peak area of echinacoside or verbascoside in the sample solution

A0: Peak area of echinacoside or verbascoside in the reference

solution

W1: The weighing amount of the sample mg

W0: the weighing amount of the reference substance mg

V1: The volume of the sample in mL

V0: volume of reference substance mL

K: Purity of reference substance

The experimental results are subject to the arithmetic mean of the parallel determination results, and the absolute difference between the two independent determination results obtained under repeatability conditions shall not exceed 10% of the arithmetic mean.

The contents of the two components are calculated and added separately. The product is calculated as a dry product, and the total content of Acteoside and echinacoside shall not be less than 20.0%.

2. Fingerprint method of *Cistanche deserticola Ma* extract

2.1 Instruments and equipment

High performance liquid chromatograph Shimadzu LC-20A

Electronic balance 1/100,000 analytical balance (Mettler Toledo MS105DU)

2.2 Reagents and materials

Acetonitrile (Fisher chromatographic purity), water (Watsons distilled water); microporous filter membrane (BOJIN nylon 0.22 μm), syringe (1 mL of Jiangxi Qingshantang Medical Equipment)

2.3 Reference chromatographic conditions

Shimadzu InertSustain AQ-C18 (4.6×250 mm, 5 μm)

2.4 Chromatographic conditions and system adaptability test

Use octadecylsilane-bonded silica gel as filler; use acetonitrile as mobile phase A and aqueous solution as mobile phase B, and perform gradient elution as specified in the following table; detection wavelength is 0-20min 280nm, 20-65min 330nm.

Time (minutes)	Mobile phase A (%)	Mobile phase B (%)
0.00～5.00	5	95
5.00～7.00	5→19	95→81
7.00～22.00	19→30	81→70
22.00～27.00	30→36	70→64
27.00～52.00	36→46	64→54
52.00～60.00	46→95	5→54
60.00～65.00	95	5

\

2.5 Reference substance map

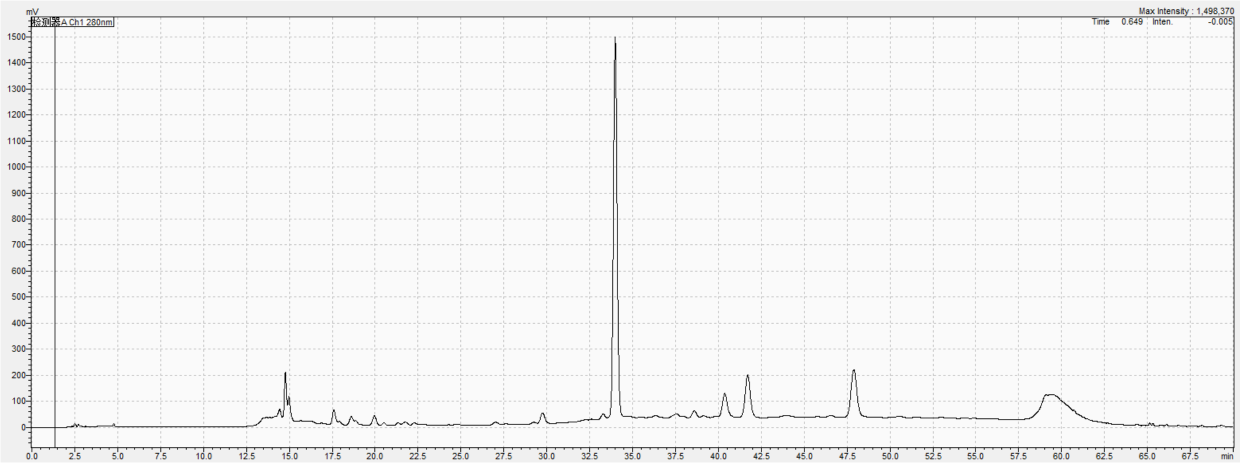

Supplement: Supplementary file 3 [file DataSheet2.ZIP › Cistanche deserticola Ma extrcts.pdf]
