# Supplementary material for: Chinese Herbal Extracts Exert Neuroprotective Effect in Alzheimer’s Disease Mouse Through the Dopaminergic Synapse/Apoptosis Signaling Pathway
Source: Front Pharmacol. 2022 Feb 28;13:817213. doi: 10.3389/fphar.2022.817213 (PMC8918930; doi:10.3389/fphar.2022.817213)
Supplement: Supplementary file 3 [file DataSheet2.ZIP › Curcuma longa L. extracts.pdf]

Measurement of the content of the curcumin and total curcumin in turmeric extract

1. Apparatus and equipment

High performance liquid chromatograph (HPLC) Shimadzu LC-20A

Electronic balance 1/100,000 analytical balance (Mettler Toledo MS105DU)

2. Reagents and materials

Acetonitrile (Fisher chromatographic purity), methanol (Fisher chromatographic purity), glacial acetic acid (Chemical reagent analysis grade of Sinopharm Group), water (Watsons distilled water); curcumin reference substance (China Institute for Food and Drug Control 20 mg), demethoxy Curcumin (Shanghai origin leaf 20 mg), bisdemethoxy curcumin (Shanghai origin leaf 20 mg); microporous filter membrane (BOJIN nylon 0.22 μm), syringe (Jiangxi Qingshantang Medical Device 1 mL)

3. Reference chromatographic conditions

Shimadzu InertSustain AQ-C18 (4.6×250 mm, 5 μm)

4. Chromatographic conditions and system adaptability test

Acetonitrile-4% glacial acetic acid aqueous solution (48:52 V/V) is the mobile phase; the flow rate is 1.0 mL/min; the detection wavelength is 430 nm; the number of theoretical plates should not be less than 4000 based on the curcumin peak.

5. Preparation of reference solution

Accurately weigh 10 mg of curcumin reference substance, 5 mg of demethoxy curcumin reference substance, and 5 mg of bis-demethoxy curcumin reference substance in a 50 mL volumetric flask, add about 20 mL of methanol to dissolve, and place it until at room temperature, dilute to volume with methanol and shake well to obtain the mother liquor (curcumin 0.2 mg/mL, demethoxycurcumin 0.1 mg/mL, bisdemethoxycurcumin 0.1 mg/mL). Prepare 6 concentrations of standard solution in sequence by the 2-fold dilution method to obtain.

6. Preparation of test solution

Accurately weigh 15 mg of the test product, add methanol to a 10ml volumetric flask and shake it up, then take 0.5ml into a 10ml volumetric flask, add methanol to the volume to the mark, shake it up, and filter to get it.

7. Determination

Precisely draw 10 μl each of the reference solution and the test solution, respectively inject the samples for determination, and inject them into the liquid chromatograph for determination.

The experimental results are subject to the arithmetic mean of the parallel determination results, and the absolute difference between the two independent determination results obtained under repeatability conditions shall not exceed 10% of the arithmetic mean.

This product is calculated as a dry product, and the content of curcumin shall not be less than 70.0%; the three types of curcumin shall not be less than 95.0%.
