# Supplementary material for: Chinese Herbal Extracts Exert Neuroprotective Effect in Alzheimer’s Disease Mouse Through the Dopaminergic Synapse/Apoptosis Signaling Pathway
Source: Front Pharmacol. 2022 Feb 28;13:817213. doi: 10.3389/fphar.2022.817213 (PMC8918930; doi:10.3389/fphar.2022.817213)
Supplement: Supplementary file 3 [file DataSheet2.ZIP › Gastrodia, elata Blume extracts.pdf]

1.Measurement of the Gastrodin in Gastrodia, elata Blume extract

1. 1Apparatus and equipment

High performance liquid chromatograph Shimadzu LC-20A

Electronic balance 1/100,000 analytical balance (Mettler Toledo MS105DU)

1.2 Reagents and materials

Acetonitrile (Fisher chromatographic purity), phosphoric acid (Chemical reagent analysis grade of Sinopharm Group), water (Watsons distilled water); Gastrodin reference substance (China Institute for Food and Drug Control 20 mg); Microporous filter membrane (BOJIN nylon 0.22 μm), syringe (Jiangxi Qingshantang medical equipment 1 mL)

1.3 Reference chromatographic conditions

Shimadzu InertSustain AQ-C18 (4.6×250 mm, 5 μm)

1.4 Chromatographic conditions and system adaptability test

Use octadecylsilane-bonded silica gel as filler; use acetonitrile-0.05% phosphoric acid solution (3:97) as mobile phase; detection wavelength is 220nm. The number of theoretical plates should not be less than 5000 calculated according to the peak of gastrodin.

1.5 Preparation of reference solution

Take an appropriate amount of gastrodin reference substance, accurately weigh it, add acetonitrile-0.05% phosphoric acid water (3:97) mixed solution to make a mixed solution containing 0.50mg gastrodin per 1ml

as mother liquor, and dilute it to 6 concentrations by the 2-fold dilution method The solution is ready.

1.6 Preparation of test solution

Take about 0.1g of this product powder (pass the No. 3 sieve), accurately weigh it, put it in a 10ml volumetric flask, add acetonitrile-0.05% phosphoric acid water (3:97) mixed solution, ultrasonic treatment (power 120W, frequency 40kHz) 30 Let it cool for a minute, dilute to the mark with a mixed solution of acetonitrile-0.05% phosphoric acid water (3:97), shake well, and filter to get it.

1.7 Measurement method

Precisely draw 10μl each of the reference solution and the test solution, and inject them into the liquid chromatograph for determination.

The experimental results are subject to the arithmetic mean of the parallel determination results, and the absolute difference between the two independent determination results obtained under repeatability conditions shall not exceed 10% of the arithmetic mean.

This product is calculated as a dry product, and the total content of gastrodin (C13H18O7) shall not be less than 1.0%.

2.Fingerprint method of Gastrodin extract

2.1 Apparatus and equipment

High performance liquid chromatograph Shimadzu LC-20A

Electronic balance 1/100,000 analytical balance (Mettler Toledo

MS105DU)

2. 2 Reagents and materials

Acetonitrile (Fisher chromatographic purity), water (Watsons distilled water); microporous filter membrane (BOJIN nylon 0.22 μm), syringe (1 mL of Jiangxi Qingshantang Medical Equipment)

2.3 Reference chromatographic conditions

Shimadzu InertSustain AQ-C18 (4.6×250 mm, 5 μm)

2.4 Chromatographic conditions and system adaptability test

Use octadecylsilane-bonded silica gel as filler; use acetonitrile as mobile phase A and aqueous solution as mobile phase B, and perform gradient elution as specified in the following table; detection wavelength is 0~35min 220nm, 35~65min 295nm .

Time (minutes)	Mobile phase A (%)	Mobile phase B (%)
0.00～18.00	2	98
18.00～22.00	2→10	98→90
22.00～32.00	10	90
32.00～45.00	10→25	90→75
45.00～60.00	25→95	75→5
60.00～65.00	95	95

2.5 Reference substance map

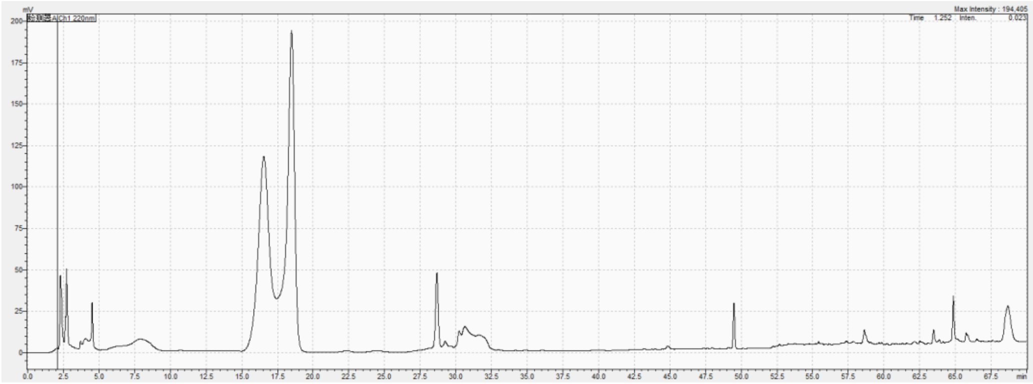

mV
检测器A Ch1 220nm
Max Intensity : 194,405
Time 1.252 Inten. 0.023
200
175
150
125
100
75
50
25
0
0.0 2.5 5.0 7.5 10.0 12.5 15.0 17.5 20.0 22.5 25.0 27.5 30.0 32.5 35.0 37.5 40.0 42.5 45.0 47.5 50.0 52.5 55.0 57.5 60.0 62.5 65.0 67.5 min
