# Supplementary material for: Chinese Herbal Extracts Exert Neuroprotective Effect in Alzheimer’s Disease Mouse Through the Dopaminergic Synapse/Apoptosis Signaling Pathway
Source: Front Pharmacol. 2022 Feb 28;13:817213. doi: 10.3389/fphar.2022.817213 (PMC8918930; doi:10.3389/fphar.2022.817213)

1.Measurement of the content of the 3, 6'-Disinapoly sucrose in Polygala tenuifolia Willd

1.1 Apparatus and equipment

High performance liquid chromatograph Shimadzu LC-20A Electronic balance 1/100,000 analytical balance (Mettler Toledo MS105DU)

2. Reagents and materials

Acetonitrile (Fisher chromatographic purity), phosphoric acid (Chemical reagent analysis grade of Sinopharm Group), water (Watsons distilled water); 3, 6'-Disinapoly sucrose (Shanghai Yuan ye Biotechnology Co., Ltd. 20 mg); Microporous filter membrane (BOJIN nylon 0.22) μm), syringe (1 mL of Jiangxi Qingshantang medical equipment)

3. Reference chromatographic conditions

Shimadzu InertSustain AQ-C18 (4.6×250 mm, 5 μm)

4. Chromatographic conditions and system adaptability test

Use octadecylsilane-bonded silica gel as filler; use acetonitrile-0.05% phosphoric acid solution (18:82) as mobile phase; detection wavelength is 320nm. The number of theoretical plates is 3, 6'-Disinapoly sucrose

The peak calculation should not be less than 3000.

5. Preparation of reference solution

Take an appropriate amount of 3, 6'-Disinapoly sucrose reference substance, accurately weigh it, add methanol solution to make a solution containing 1mg per 1ml, and prepare 6 concentrations of standard

solution sequentially by the 2-fold dilution method to obtain.

6. Preparation of test solution

Precisely weigh 1g of the powder (passed through the No. 3 sieve), add 70% methanol in a 50ml volumetric flask, ultrasonic treatment (power 400W, frequency 40kHz) for 30min, let cool, use 70% methanol to the mark, shake well and filter.

7. Measurement method

Precisely draw 10 μl each of the reference solution and the test solution, respectively inject the samples for determination, and inject them into the liquid chromatograph to obtain the results.

The experimental results are subject to the arithmetic mean of the parallel determination results, and the absolute difference between the two independent determination results obtained under repeatability conditions shall not exceed 10% of the arithmetic mean.

This product is calculated as dry product and contains 3, 6'-Disinapoly sucrose, not less than 1.0%.

2. Fingerprint method of Polygala extract

2.1 Apparatus and equipment

High performance liquid chromatograph Shimadzu LC-20A

Electronic balance 1/100,000 analytical balance (Mettler Toledo MS105DU)

2. 2 Reagents and materials

Acetonitrile (Fisher chromatographic purity), water (Watsons distilled water); microporous filter membrane (BOJIN nylon 0.22 μm), syringe (1 mL of Jiangxi Qingshantang Medical Equipment)

2.3 Reference chromatographic conditions

Shimadzu InertSustain AQ-C18 (4.6×250 mm, 5 μm)

2.4 Chromatographic conditions and system adaptability test

Time (minutes)	Mobile phase A (%)	Mobile phase B (%)
0.00～5.00	5	95
5.00～8.00	5→8	95→92
8.00～11.00	8	92
11.00～17.00	8→13	92→87
17.00～23.00	13	87
23.00～26.00	13→17	87→83
26.00～30.00	17	83
30.00～31.00	17→21.5	83→78.5
31.00～73.00	21.5→31	78.5→69
73.00～93.00	31→50	69→50
93.00～103.00	50→60	50→40
103.00～108	60→70	40→30

108.00～110.00	70→100	30→10

Use octadecylsilane-bonded silica gel as filler; use acetonitrile as mobile phase A and aqueous solution as mobile phase B, and perform gradient elution as specified in the following table; detection wavelengths are 220 nm and 320 nm.

Reference substance map

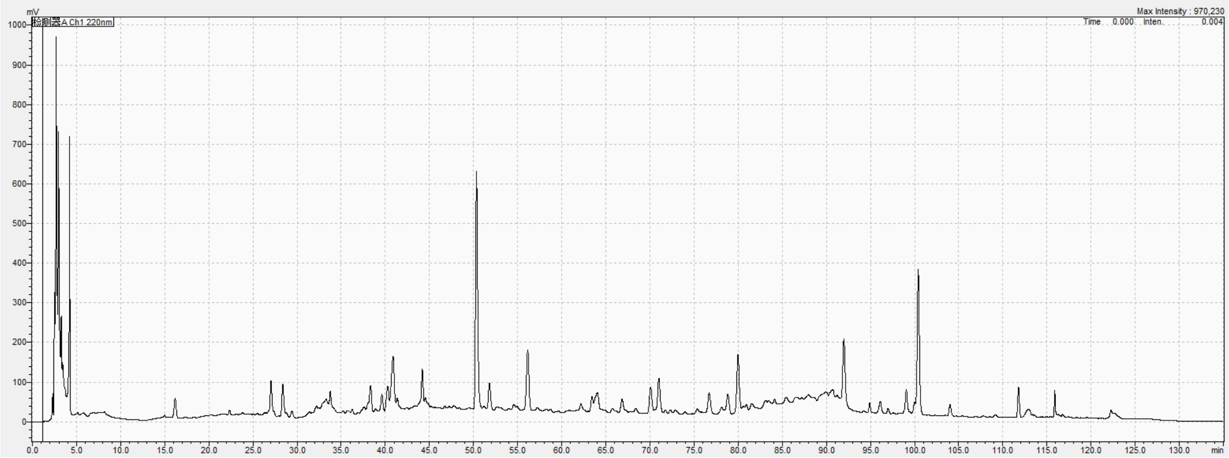

Supplement: Supplementary file 3 [file DataSheet2.ZIP › Polygala tenuifolia Willd extracts.pdf]
