# Supplementary material for: Chinese Herbal Extracts Exert Neuroprotective Effect in Alzheimer’s Disease Mouse Through the Dopaminergic Synapse/Apoptosis Signaling Pathway
Source: Front Pharmacol. 2022 Feb 28;13:817213. doi: 10.3389/fphar.2022.817213 (PMC8918930; doi:10.3389/fphar.2022.817213)

Rehmannia lutinosa (Gaertn.) DC **extract fingerprinting method**

1. Apparatus and equipment

High performance liquid chromatograph Shimadzu LC-20A

Electronic balance 1/100,000 analytical balance (Mettler Toledo MS105DU)

2. Reagents and materials

Acetonitrile (Fisher chromatographic purity), water (Watsons distilled water); microporous filter membrane (BOJIN nylon 0.22 μm), syringe (1 mL of Jiangxi Qingshantang Medical Equipment)

3. Reference chromatographic conditions

Shimadzu InertSustain AQ-C18 (4.6×250 mm, 5 μm)

4. Chromatographic conditions and system adaptability test

Use octadecylsilane-bonded silica gel as filler; use acetonitrile as mobile phase A and aqueous solution as mobile phase B, and perform gradient elution as specified in the following table; detection wavelength is 0-25min 280nm, 25-70min 330nm .

Time (minutes)	Mobile phase A (%)	Mobile phase B (%)
0.00～5.00	5	95
5.00～17	5→20	80
17.00～24.00	20→30	70
24.00～27.00	30→36	70→64
27.00～50.00	36→45	64→55

50.00～53.00	45→60	55→40
53.00～70.00	60→95	40→5

Reference substance map

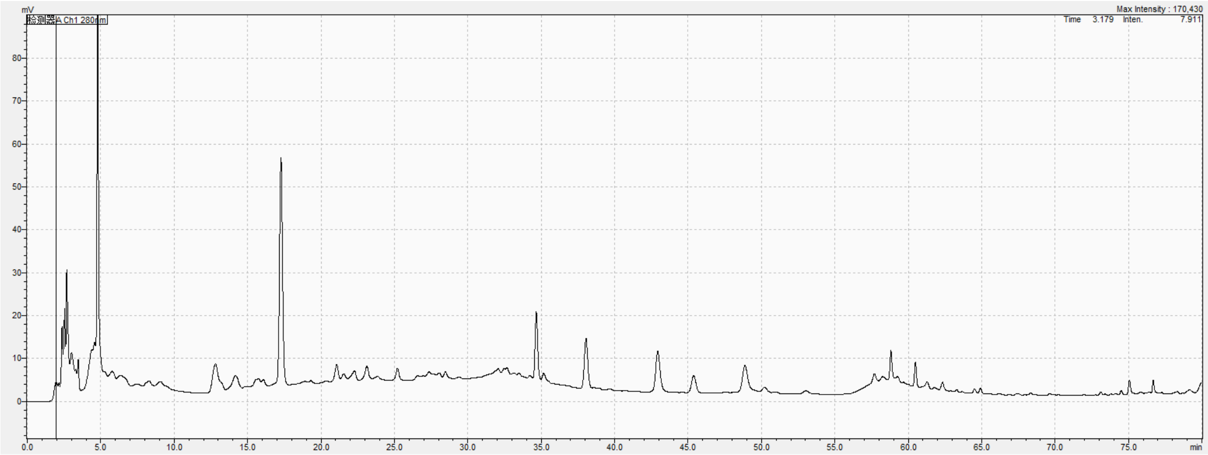

Supplement: Supplementary file 3 [file DataSheet2.ZIP › Quality control of the Rehmannia lutinosa (Gaertn.) DC extract.pdf]
